# Increased spontaneous physical activity in female MEST-deficient mice protects against diet-induced obesity

**DOI:** 10.3389/fendo.2025.1680158

**Published:** 2025-10-29

**Authors:** Rea Victoria P. Anunciado-Koza, M. Elena Martinez, Victoria DeMambro, Arturo Hernandez, Robert A. Koza

**Affiliations:** ^1^ Center for Molecular Medicine, MaineHealth Institute for Research, Scarborough, ME, United States; ^2^ Graduate School of Biomedical Sciences and Engineering, University of Maine, Orono, ME, United States; ^3^ Department of Medicine, Tufts University School of Medicine, Boston, MA, United States; ^4^ Pennington Biomedical Research Center, Baton Rouge, LA, United States

**Keywords:** obesity, adipose tissue, hypothalamus, epigenetics, mouse models, physical activity, thyroid hormones

## Abstract

**Introduction:**

Mesoderm-specific transcript (*Mest*), a paternally expressed imprinted gene, is involved in the modulation of adipose tissue expansion. *Mest* is also highly expressed in the developing and adult brain, suggesting a role in behavioral phenotypes. Previously, we showed that female mice with paternal *Mest* inactivation (*Mest* ^pKO^) exhibit no discernible behavioral impairments compared to wild-type mice. In this study, we performed metabolic phenotyping of female *Mest* ^pKO^ mice in response to a dietary challenge.

**Methods:**

Eight-week-old female and male wild-type and *Mest* ^pKO^ mice were fed a control or Western diet (40 kcal% fat) until 24 weeks of age. Body weight, body composition, and metabolic parameters were measured during the course of the feeding regimens, and gene expression and type-2-deiodinase (DIO2) activity were examined in white adipose tissue and brain at the end of the study.

**Results:**

*Mest* ^pKO^ female mice fed a Western diet were protected against diet-induced obesity. Strikingly, these mice showed increased ambulatory activity and speed, coupled with reduced resting parameters, suggesting a role for MEST in the regulation of spontaneous physical activity, a form of nonexercise activity thermogenesis. When considering body mass (control diet) and lean mass (Western diet), energy expenditure was increased in the female *Mest* ^pKO^ mice. Male *Mest* ^pKO^ mice did not exhibit these changes. Analyses of hypothalamic gene expression revealed upregulation of *Dio2*, and RNA-seq highlighted differential expression of numerous thyroid hormone-responsive genes in *Mest* ^pKO^ female mice.

**Conclusion:**

Mechanistically, our results suggest that MEST directly or indirectly regulates thyroid hormone-responsive genes in the hypothalamus, thereby modulating the neurobiological control of nonexercise activity thermogenesis in Western diet-fed female mice.

## 1 Introduction

The global increase in the prevalence of obesity substantially raises the risk of its related comorbidities, such as type 2 diabetes, metabolic dysfunction-associated steatotic liver disease (MASLD), hypertension, cardiovascular disease, dementia, joint pain, and various cancers, imposing a significant burden on public health (1, 2). The development of obesity results from a combination of genetic, environmental, lifestyle, and epigenetic factors (3). Although monozygotic twin studies have revealed that the heritability of obesity is more than 40%, the identification of > 1,000 obesity-linked genetic variants through genome-wide association studies still accounts for a small fraction of the variance of body mass index (4, 5). A significant component of obesity remains unaccounted for, further reinforcing the complexity of the condition.

Mesoderm-specific transcript (MEST), a paternally expressed imprinted gene with an unknown molecular function, belongs to the alpha/beta hydrolase protein family, which exhibits a broad range of activities (6, 7). *Mest* mRNA and protein levels vary greatly—up to ~ 50-fold—in white adipose tissue (WAT) from individual C57BL/6J mice fed an obesogenic diet, and variations in WAT *Mest* show a positive association with fat mass deposition (8). The role of MEST in adipose tissue has been well characterized (9–11). Transgenic overexpression of *Mest* in mice results in enlarged adipocytes (10), whereas mice with global and adipocyte-specific inactivation of *Mest* show reduced diet-induced obesity (12), further highlighting a role for *Mest* in WAT expansion. Moreover, *Mest* is localized to the endoplasmic reticulum of adipocytes, in close proximity to the membranes of lipid droplets (13).

Human total daily energy expenditure is composed of basal metabolic rate, the thermic effect of food, and activity thermogenesis, which includes exercise and “nonexercise activity thermogenesis” (NEAT) (14). Purposeful human exercise is volitional, whereas NEAT encompasses all activities outside of chosen exercise (15), including the drive to stand and walk, fidgeting, and gesticulating (16). NEAT-associated activities can account for 100 to 800 kcal of energy expenditure (EE) per day (17). Harnessing NEAT as a mechanism to increase total daily energy expenditure is an attractive strategy to dissipate energy and provide protection against diet-induced obesity during times of excess caloric intake. Weight gain in obesity results from a chronic imbalance between caloric intake and energy expenditure. Excessive caloric intake combined with physical inactivity contributes to the development of obesity. Increasing physical activity through NEAT represents a potential strategy to enhance the energy expenditure component of body weight regulation (18, 19).

The significance of MEST in the central nervous system is highlighted by its expression in the embryonic midbrain, as well as in the early postnatal and adult brain (20–22). *Mest* is one of the most highly upregulated genes during early mesodiencephalic dopaminergic neuronal development in the midbrain (22) and during the morphological transition of primary neurons during formation of the mammalian neocortex (23). Mice with inactivation of *Mest* (*Mest* ^tm1.2Rkz^) generated in our laboratory show no discernible impairments in object recognition memory, social behavior, or maternal behavior (20). Paradoxically, abnormal maternal behavior (21) and reduced climbing behavior (22) were reported by others using a different *Mest* knockout mouse (*Mest* ^tm1Masu^). Phenotypic differences between these two models may result from differences in genetic background or gene-targeting approaches (20).

This study focused on metabolic phenotyping of female mice with paternal inactivation of *Mest* (*Mest* ^pKO^) fed a control or obesogenic diet. Remarkably, female *Mest* ^pKO^ mice exhibited increased spontaneous physical activity (SPA) and were protected against diet-induced obesity. RNA-sequencing (RNA-seq) analysis of the hypothalamus from MEST-deficient female mice revealed upregulation of several thyroid hormone (TH)-responsive genes, suggesting a mechanism involving TH and MEST in the neurobiological regulation of increased SPA and protection against obesity in female *Mest* ^pKO^ mice.

## 2 Materials and methods

### 2.1 Animals, diets, and experimental design

**Figure 1:**
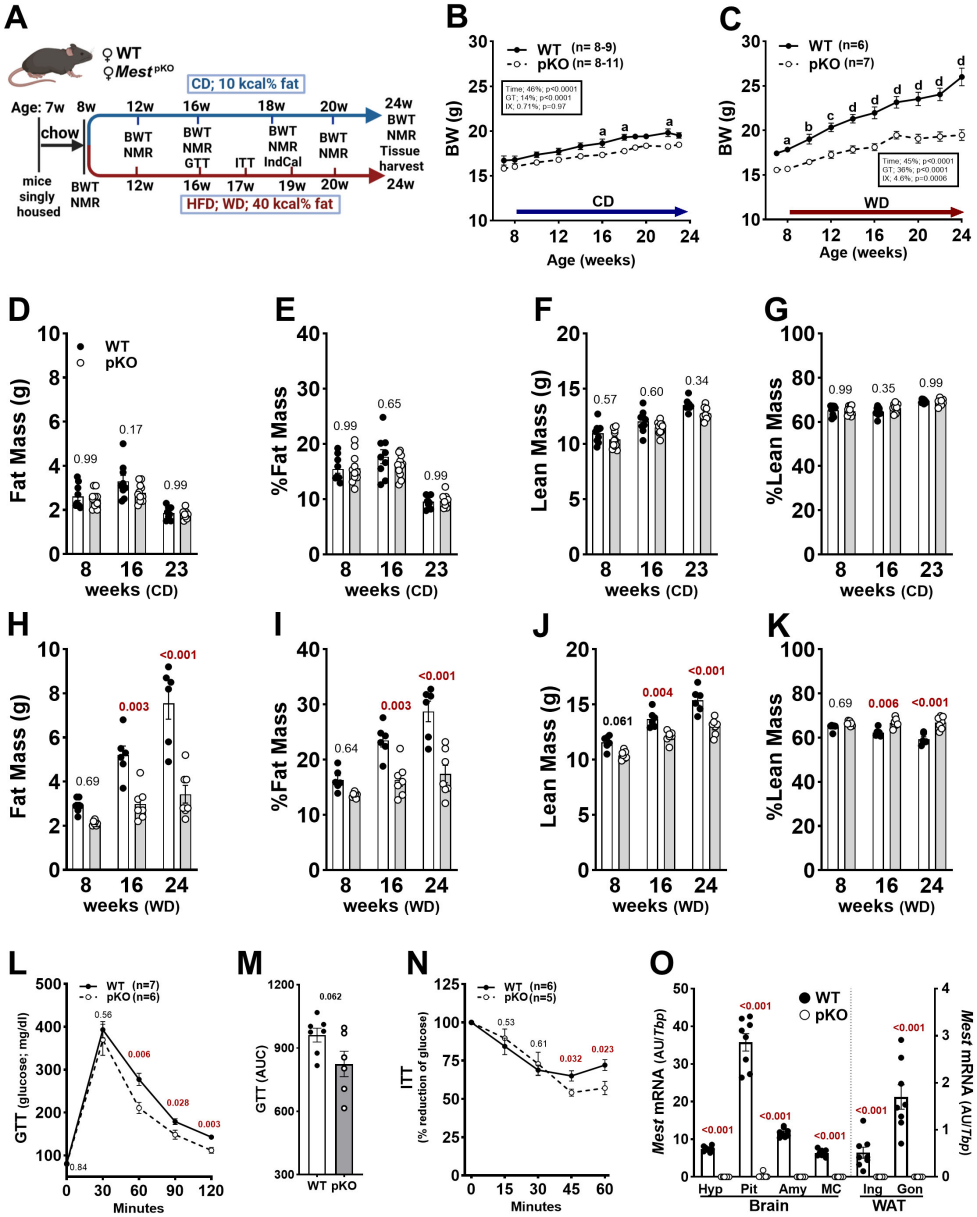
Phenotypic analyses of WT and *Mest* ^pKO^ (pKO) female mice fed control (CD) and Western diet (WD). **(A)** Schematic showing the study design used for dietary studies. BWs of WT and *Mest* ^pKO^ female mice fed CD **(B)** and WD **(C)** from 8 to 24 weeks of age. **(D)** Fat mass, **(E)** % fat mass, **(F)** lean mass, and **(G)** % lean mass of CD-fed female WT and *Mest* ^pKO^ mice at 8, 16, and 23 weeks of age measured by NMR. **(H)** Fat mass, **(I)** % fat mass, **(J)** lean mass, and **(K)** % lean mass of WD-fed female mice at 8, 16, and 24 weeks of age. **(L)** Glucose tolerance test (GTT) and **(M)** area under the curve (AUC) for WT and *Mest* ^pKO^ female mice fed WD at 16 weeks of age. **(N)** Insulin tolerance test of WD-fed WT and *Mest* ^pKO^ female mice at 17 weeks of age. **(O)** Analyses of *Mest* gene expression show it to be almost completely inactivated in the hypothalamus (Hyp), pituitary (Pit), amygdala (Amy), motor cortex (MC), and inguinal (Ing) and gonadal (Gon) white adipose tissue of female *Mest* ^pKO^ mice. Data in figure panels **(B–K)** were analyzed by two-way ANOVA, and **(L–O)** by unpaired *t*-tests. Annotations with a–d indicate *p*-values lower than 0.05, 0.01, 0.001, and 0.0001 for data in **(B**, **C)**. *p*-values are numerically indicated for the remaining figures, with < 0.001 indicating a *p*-value of less than 0.0005. Schematic **(A)** was created in https://BioRender.com.

The Institutional Animal Care and Use Committee at MaineHealth Institute for Research approved all animal experiments in accordance with National Institutes of Health guidelines for the care and use of laboratory animals. We used *Mest* ^tm1.2Rkz^ mice (12) with a global inactivation of *Mest* and littermate wild-type (WT) mice for our studies. All mice were congenic on the C57BL/6J genetic background. Mice were fed the 2018 Teklad Global 18% protein rodent diet (Envigo, Indianapolis, IN, USA) and housed in a barrier facility with standard light and humidity conditions at an ambient temperature of 23 °C–24 °C. For dietary studies, female mice were fed a control (CD; D14042701i, Research Diets, New Brunswick, NJ, USA) or a Western diet (WD; 40 kcal% fat; D12079Bi, Research Diets, New Brunswick, NJ, USA). Singly housed 8-week-old, age-matched female and male WT mice and *Mest* ^pKO^ mice were fed a CD or WD until 24 weeks of age, as illustrated in **Figure 1A**. Body weight (BW), body composition (NMR), glucose and insulin tolerance, and metabolic parameters (indirect calorimetry) were measured as indicated (**Figure 1A**). Detailed analyses were performed on mutant female mice that exhibited a pronounced increase in SPA. Male data are presented in the **Supplementary Material** as indicated in the Results section.

### 2.2 Body composition and metabolic cage analyses

Body composition of mice was measured using a Minispec LF50 TD-NMR analyzer (Bruker, Massachusetts) as previously described (12). Indirect calorimetry was performed using the Promethion metabolic cage system (Sable Systems International, North Las Vegas, NV, USA). Metabolic variables—oxygen consumption (VO_2_), carbon dioxide production (VCO_2_), EE, respiratory exchange ratio (RER), and locomotor activity—were analyzed using web-based CalR software (24). Generalized linear modeling, using BW, fat mass (FM), and lean mass (LM) as covariates, was performed for VO_2_, VCO_2_, and EE. Analysis of variance (ANOVA) was used for mass-independent variables, including locomotor activity and RER. Fine movements, such as grooming and scratching, were calculated as the difference between AllMeters and PedMeters (Sable Systems). NEAT was calculated as previously described (25). Total EE (TEE; kcal/h) was divided by two to obtain TEE values every 30 min (TEE-30). The lowest TEE-30 value for each day was considered the basal metabolic rate (BMR). NEAT was then calculated by subtracting BMR from each TEE-30, yielding NEAT values every 30 min (NEAT-30) for each day.

### 2.3 Glucose and insulin tolerance tests

Glucose tolerance tests (GTT) were performed after an overnight fast, followed by intraperitoneal administration of 2 g/kg glucose. Insulin tolerance tests (ITT) were performed after a 4-h fast, with intraperitoneal administration of 0.5 IU/kg insulin (Humulin R U-100, Eli Lilly and Company, Indianapolis, IN, USA). Blood glucose levels in the tail vein were monitored using a glucometer (Accu-check Aviva Plus, Roche Diagnostics, Indianapolis, IN, USA).

### 2.4 Serum corticosterone assay

At the end of the study, blood was collected from isoflurane-anesthetized mice by intracardiac puncture into BD SST™ tubes (Becton Dickinson, Franklin Lakes, NJ, USA). The tubes were inverted 10 × and allowed to clot at room temperature for 30 min. Samples were centrifuged at 1,300×*g* for 15 min, and serum was collected and stored at − 70 °C. Serum corticosterone levels were measured using the Corticosterone AssayMax ELISA Kit (EC3001-1, AssayPro, St. Charles, MO, USA).

### 2.5 DIO2 and DIO3 enzymatic assays

Type-2-deiodinase (DIO2) and type-3-deiodinase (DIO3) enzymatic activities were determined as previously described (26). In brief, frozen tissues were homogenized on ice in a buffer containing 20 mM Tris-HCl, 0.25 M sucrose, and 5 mM dithiothreitol (Sigma-Aldrich, St. Louis, MO, USA) (pH = 7.0 for DIO2 and 7.4 for DIO3). A suitable volume of tissue homogenate containing 50–200 μg of protein was used in the enzymatic reaction to ensure that total deiodination did not exceed 40% and remained proportional to the protein content. Tissue homogenates were incubated at 37 °C for an hour in the presence of 25 mM dithiothreitol (Sigma-Aldrich) with either 1 nM of ^125^I-labeled thyroxine (T4; Catalog No. NEX111X, Revvity, Waltham, MA, USA) for the DIO2 assay, or 2 nM ^125^I-labeled triiodothyronine (T3; Catalog No. NEX110X, Revvity) for the DIO3 assay. Unlabeled T3 (1 μM) was added to the DIO2 enzymatic reaction to prevent interference from DIO3 in the DIO2 assay. The enzymatic reactions were stopped with 1 vol of ethanol, and an aliquot was subjected to paper chromatography (Catalog No. 3001-614, Whatman, GE Healthcare Life Sciences, Chicago, IL, USA) as previously described (27), using ammonia-saturated 2-methyl-2-butanol as the eluent. Chromatograms were autoradiographed, and the bands corresponding to different metabolites were excised and counted using a gamma counter. Deiodination was determined based on the percentage of ^125^I-T3 produced from T4 (for DIO2 assay), accounting for unlabeled T3, or the percentage of ^125^I-3,3′-diiodothyronine produced from T3 (for the DIO3 assay).

### 2.6 Adipose tissue histology

Subcutaneous inguinal (iWAT) and gonadal white adipose tissue (gWAT) were fixed in Bouin’s solution (Harleco, Millipore Sigma, Darmstadt, Germany) for 48 h, paraffin-embedded, and processed for H&E staining.

### 2.7 RNA isolation and quantitative reverse transcription PCR

Tissue total RNA was extracted as previously described (12) using the RNeasy Mini Kit (Qiagen, Germantown, MD, USA). Briefly, tissues were homogenized in TriReagent (Molecular Research Center, Cincinnati, OH, USA), and RNA was purified using the RNeasy Mini Kit and RNAse-free DNAse (Qiagen). One-step quantitative reverse transcription PCR (RT-qPCR) was performed using the TaqMan RNA-to-CT Reagent (Thermo Fisher Scientific, Waltham, MA, USA) on a CFX384 Real-Time Platform (BioRad, Hercules, CA, USA). Two-step RT-qPCR was performed using SYBR Select Master Mix (Thermo Fisher Scientific) with cDNA synthesized using the High-Capacity cDNA Reverse Transcription Kit (Thermo Fisher Scientific). Gene-specific TaqMan probes (LGC, BioSearch Technologies, Middlesex, TW11 0LY, UK) and PrimeTime primers (Integrated DNA Technologies, Coralville, IA, USA), as well as primer/probe combinations, were used for gene quantification. Gene expression was normalized to TATA box binding protein (*Tbp*) for TaqMan assays. The relative abundance of genes of interest measured using SYBR green was quantified and normalized to *Tbp* using the 2-ΔΔCT method. Probe and primer sequences for TaqMan assays, primer sequences for SYBR green assays, and stock numbers for primer and probe sets (Integrated DNA Technologies, Coralville, IA, USA) are listed in **Supplementary File S1**.

### 2.8 RNA-sequencing

Bulk RNA-seq of the whole mouse hypothalamus was performed at the Pennington Biomedical Research Center Genomics Core Facility (Baton Rouge, LA, USA). Total RNA quality was assessed using the Agilent Bioanalyzer RNA 6000 Assay. RNA-seq library construction was performed using the Lexogen QuantSeq 3’ mRNA-seq V2 Library Prep Kit FWD with UDI (Catalog No. 191.96; Lexogen, Greenland, NH, USA). Libraries were validated using the Agilent BioAnalyzer High Sensitivity DNA Assay (Agilent, Santa Clara, CA, USA). The ~ 275-bp libraries were pooled in equimolar amounts and sequenced on the Illumina NextSeq2000 (Illumina, San Diego, CA, USA) at 75 bp read length. Quality control of raw sequencing data was checked using FastQC (28), and Cutadapt (29) was used to trim adapter sequences and low-quality bases, ensuring cleaner data for downstream analysis. The cleaned reads were aligned to the reference genome (GRCm39 release 110) using the STAR aligner (30). Following alignment, HTSeq (31) was used to count the number of reads mapping to each gene, generating a count matrix. Differential analysis of RNA read count data was then performed using DESeq (32), which models the total counts as a negative binomial distribution and applies an empirical Bayes shrinkage-based method to estimate signal dispersion and fold changes. Gene expression signals were logarithmically transformed (base 2) for all downstream analyses, with the lowest expression value being set to 1. Genes with an absolute log fold change ≥ 1 and a false discovery rate (FDR) of 5% were considered differentially expressed. Analysis of differentially expressed genes was performed using the online enrichment analysis tool Enrichr (33). Gene ontology data were obtained from the GO Biological Process 2023, GO Cellular Component 2023, GO Molecular Function 2023, and MGI Mammalian Phenotype Level 4 libraries. Pathway analysis terms were retrieved from the Reactome 2022 library. Statistical significance was defined as an adjusted *p*-value ≤ 0.05.

### 2.9 Data analyses

All results are expressed as mean ± SEM. Data were tested for normality to assess Gaussian distribution, and differences between datasets were analyzed using unpaired parametric *t*-tests, one- or two-way ANOVA, or simple linear regression in GraphPad Prism 10.0.2.

## 3 Results

### 3.1 Female mice with paternal inactivation of *Mest* show resistance to diet-induced obesity

Using the experimental design in **Figure 1A**, female WT and *Mest* ^pKO^ mice were fed a CD (10 kcal% fat) or WD (40 kcal% fat) from 8 to 24 weeks of age. BW measurements showed that CD-fed *Mest* ^pKO^ female mice had slightly reduced BW compared to WT mice throughout the 8–14-week period (**Figure 1B**), due to small reductions in both FM and LM (**Figures 1D–G**). When fed WD from 8 to 24 weeks of age, mice showed considerable divergence in BW (**Figure 1C**), with WT mice gaining weight approximately twofold faster than *Mest* ^pKO^ (0.51 g/week vs. 0.24 g/week; *p* = 0.0013). Indices of FM prior to WD feeding at 8 weeks of age (**Figures 1H, I**) and average weekly WD intake measured from 14 to 16 weeks of age (WT, 2.40 g/week vs. *Mest* ^pKO^, 2.28 g/week; *p* = 0.23), showed no difference between genotypes. However, divergence in FM occurred between genotypes by 16 weeks of age (**Figures 1H, I**). Analyses using two-way ANOVA showed that time and genotype contributed to differences in FM between WT and *Mest* ^pKO^ mice fed WD (**Supplementary Table S1A**). While LM was similar between genotypes at 8 weeks of age, significant differences emerged after mice were fed WD (**Figure 1J**). Percentage LM relative to BW decreased by ~ 5%–6% in WT mice fed WD from 8 to 24 weeks of age, whereas it remained consistent in *Mest* ^pKO^ mice throughout the period and was significantly higher than in WT mice at 16 and 24 weeks of age (**Figure 1K**). Since ~ 25% of adipose tissue mass consists of fat-free mass—often referred to as the quarter fat-free mass rule (34)—our results suggest that this component contributes to overall LM in WT mice (**Figure 1J**). Additionally, anal–nasal length measurements of 24-week-old WT and *Mest* ^pKO^ female mice fed a 10-kcal% fat diet showed no differences between genotypes (WT, 91.2 mm; *Mest* ^pKO^, 90.2 mm; *n* = 8 mice per genotype; *p* = 0.20), suggesting that *Mest* inactivation does not significantly affect longitudinal growth. Glucose tolerance in CD-fed mice was comparable between genotypes (**Supplementary Table S1B**); however, when fed WD, *Mest* ^pKO^ female mice exhibited significantly improved blood glucose levels after glucose injection compared to WT mice (**Figure 1L**). This was reflected by marginally significant differences (*p* = 0.062) in the area under the curve (**Figure 1M**). While circulating insulin levels were not measured in our study, an insulin tolerance test assessing whole-body insulin action showed improved insulin sensitivity in *Mest* ^pKO^ female mice compared to WT (**Figure 1N**).

Male *Mest* ^pKO^ mice fed CD or WD from 8 to 16 weeks of age showed significantly reduced BW gain associated with LM compared to WT mice (**Supplementary Figure S1**). Thus, reduced BW gain in response to dietary intervention is a phenotype consistent across both sexes.

Inactivation of the paternal allele of *Mest* has been shown to ablate *Mest* in WAT (12). In this study, we demonstrate similar monoallelic expression of *Mest* in various regions of the brain, as evidenced by inactivation of *Mest* in the hypothalamus (Hyp), pituitary (Pit), amygdala (Amy), and motor cortex (MC) of *Mest* ^pKO^ mice (**Figure 1O**).

### 3.2 Female *Mest* ^pKO^ mice fed a Western diet show increased energy expenditure and physical activity compared to WT mice

**Figure 2:**
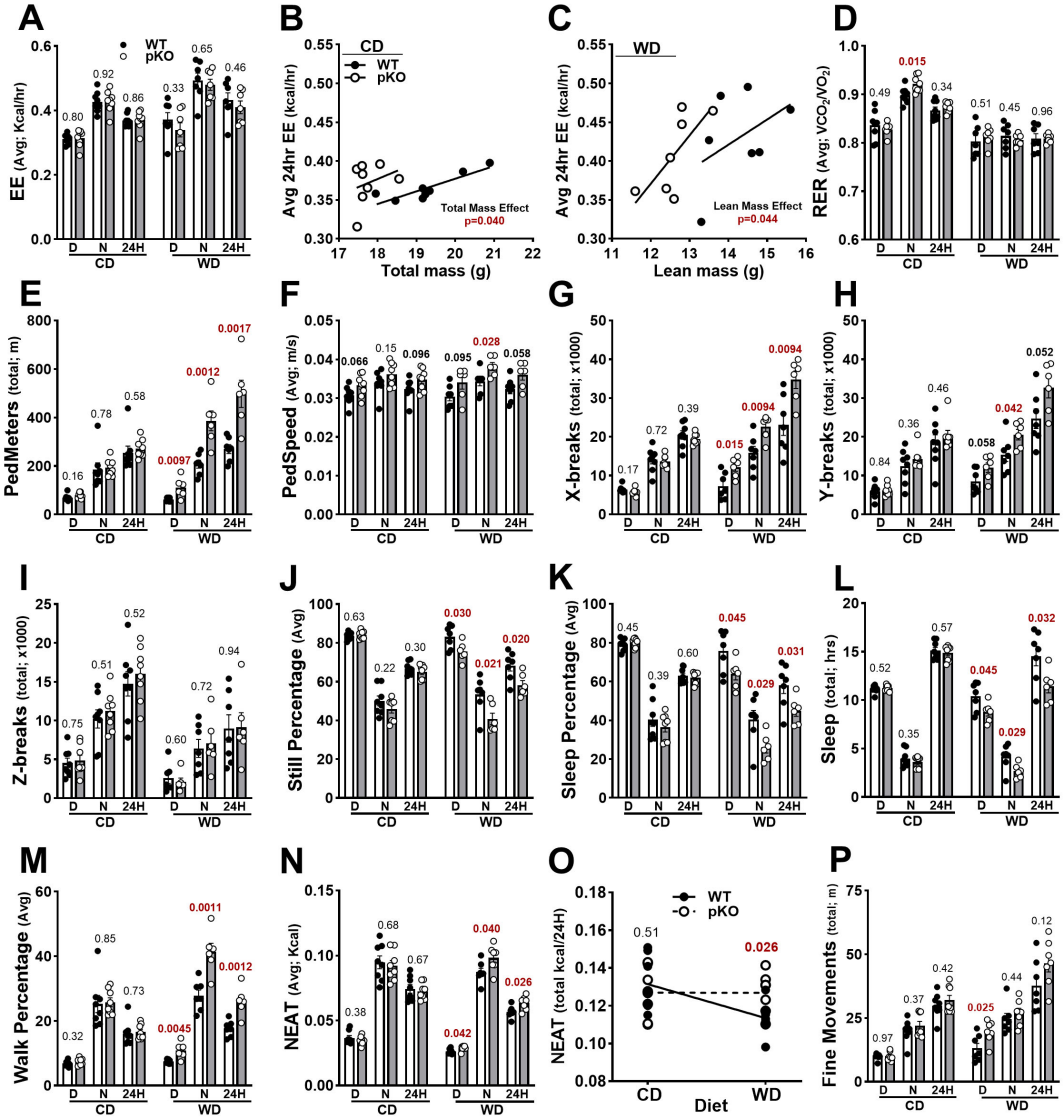
Metabolic and physical activity measurements of female WT and *Mest* ^pKO^ (pKO) mice fed CD or WD. **(A–C)** Energy expenditure (EE), **(D)** respiratory exchange ratio (RER), and **(E**–**M)** indices of activity and sleep were measured in female WT and *Mest* ^pKO^ mice fed CD or WD for 16 weeks. **(N**, **O)** Nonexercise activity thermogenesis (NEAT) and **(P)** fine movements were calculated as described in **Supplementary File Methods**. Data in **(A**, **D**–**P)** were analyzed via unpaired *t*-tests for each circadian period comparing WT and *Mest* ^pKO^ female mice fed CD or WD. Data in **(B, C)** were calculated to show significant differences between genotypes when adjusted for **(B)** total mass or **(C)** lean mass. *p*-values are numerically indicated in all figures, with < 0.001 denoting a *p*-value of less than 0.0005.

Indirect calorimetry of female WT and *Mest* ^pKO^ mice fed CD or WD for 16 weeks showed no significant differences in average daily EE between genotypes (**Figure 2A**). However, analysis using generalized linear modeling (24) indicated that BW (total mass) was the primary determinant of 24-h EE between genotypes in CD-fed mice (**Figure 2B**; *p* = 0.040). The leftward shift in the regression plot for EE of *Mest* ^pKO^ mice (**Figure 2B**) indicates a significant genotype effect associated with a higher metabolic rate. Furthermore, VO_2_, VCO_2_, and EE all exhibited significant genotype effects during dark hours when BW was included as a covariate (**Supplementary Table S2A**). In mice fed WD, LM was the primary determinant of 24-h EE (**Figure 2C**; *p* = 0.044). LM was also a significant covariate for VO_2_, VCO_2_, and EE during the light hours, with no observable genotype effect (**Supplementary Table S2B**). RER (VCO_2_/VO_2_) was comparable between genotypes fed CD, except that *Mest* ^pKO^ showed significantly higher RER than WT mice during dark hours (**Figure 2D**, **Supplementary Table S2A**), suggesting increased carbohydrate metabolism at night. No differences in RER were observed between WT and *Mest* ^pKO^ female mice fed WD (**Figure 1D**, **Supplementary Table S2B**).

Average ambulatory distance (**Figure 2E**) and speed within the cage (**Figure 2F**) were consistent between *Mest* ^pKO^ and WT female mice fed CD. Generalized linear modeling using one-way ANOVA for mass-independent variables determined significant genotype effects for pedestrian locomotion and total distance in the cage for all time periods in *Mest* ^pKO^ (**Supplementary Table S2A**). When fed WD, *Mest* ^pKO^ female mice showed significantly increased pedestrian distance during the day, night, and 24 h compared to WT (**Figure 2E**, **Supplementary Table S2B**). Average pedestrian speed was significantly increased for *Mest* ^pKO^ female mice compared with WT female mice fed WD only at night (**Figure 2F**). Measurements of X- and Y-breaks showed no differences between genotypes when fed CD but were significantly increased in *Mest* ^pKO^ mice during all time periods compared to WT when fed WD (**Figures 2G, H**). No differences in Z-breaks were observed between WT and *Mest* ^pKO^ female mice fed either CD or WD (**Figure 2I**). Measurements of % time spent still or asleep, and total sleep hours, showed no differences between genotypes fed CD but significant differences in mice fed WD, with *Mest* ^pKO^ mice showing reduced still and sleep percentages and times (**Figures 2J–L**). Correspondingly, the time spent walking was also significantly increased for all time periods, only for *Mest* ^pKO^ female mice compared to WT female mice when fed WD (**Figure 2M**).

In the context of dietary fat, *Mest* ^pKO^ female mice show increased SPA compared to WT mice, including pedestrian locomotion (PedMeters), X- and Y-breaks, and time spent walking (**Figures 2E, G, H, M**), which correlate with reduced time spent still and sleeping (**Figures 2J–L**). The energy associated with SPA, referred to as NEAT, shows no differences between genotypes when fed CD but is higher in the *Mest* ^pKO^ compared to WT at all time periods when fed WD (**Figure 2N**). Further analysis revealed that WT mice show reduced NEAT when fed WD compared to *Mest* ^pKO^, whereas both genotypes maintained comparable levels of NEAT when fed CD (**Figure 2O**). Additionally, *Mest* ^pKO^ female mice fed WD were more engaged in fine movements, such as grooming and scratching, particularly during daylight, compared to WT mice when fed WD (**Figure 2P**). Thus, increased SPA and NEAT, in the context of an obesogenic diet and paternal inactivation of *Mest*, may protect *Mest* ^pKO^ female mice from diet-induced obesity. Resistance to dietary obesity in female *Mest* ^pKO^ mice, to some extent, parallels that previously observed in male mice (12); however, no changes in physical activity were observed in *Mest* ^pKO^ male mice fed CD or WD (**Supplementary Tables S3A, B**).

### 3.3 Hypothalamic gene expression is altered in control diet-fed female *Mest* ^pKO^ mice

**Figure 3:**
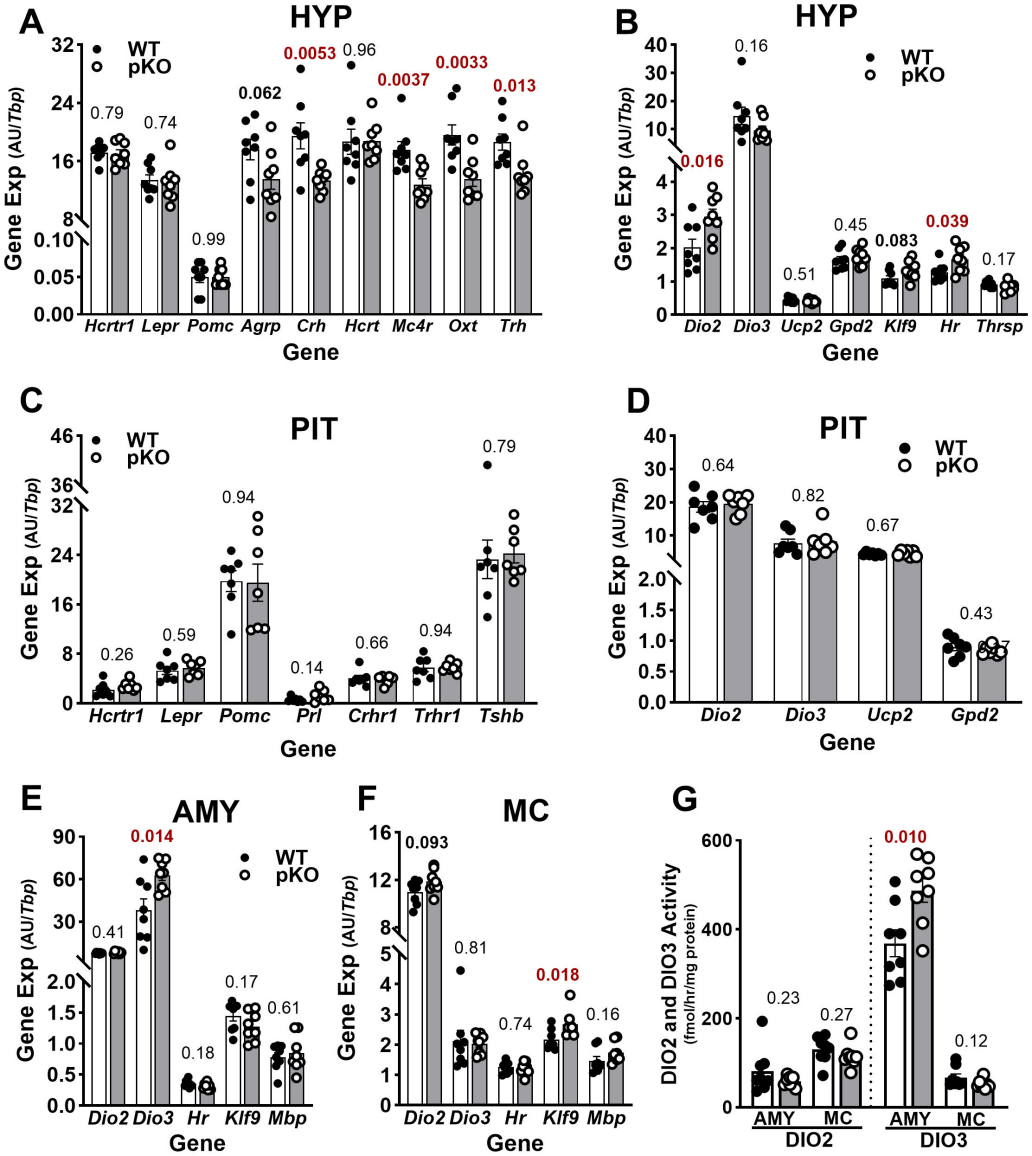
Key thyroid hormone metabolic and responsive genes show increased expression in the hypothalamus of CD-fed female *Mest* ^pko^ (pKO) mice. **(A, B)** Data show hypothalamic (Hyp), **(C, D)** pituitary (Pit), **(E)** amygdala (Amy), and **(F)** motor cortex (MC) gene expression in CD-fed WT and *Mest* ^pKO^ mice measured using RT-qPCR. **(G)** Enzymatic measurements of type 2 (DIO2) and type 3 (DIO3) deiodinases were measured in the Amy and MC from female WT and *Mest* ^pKO^ mice fed CD. Unpaired *t*-tests were used to compare gene expression data between genotypes, and *p*-values are numerically in all figures.

Analysis of *Mest* mRNA in multiple brain regions of WT and *Mest* ^pKO^ mice, including the hypothalamus, pituitary, amygdala, and motor cortex, confirms that transcription occurs exclusively from the paternal allele, with the highest expression in the pituitary (**Figure 1O**). To investigate the neurobiological basis for increased physical activity without the influence of dietary fat, several potential candidate genes were measured in the different brain regions of CD-fed WT and *Mest* ^pKO^ female mice. Initial studies focused on the hypothalamus and pituitary because of their well-recognized role in metabolism. *Mest* ^pKO^ female mice showed significantly reduced hypothalamic expression of corticotropin-releasing hormone (*Crh*), oxytocin (*Oxt*), thyrotropin-releasing hormone (*Trh*), and the primary central melanocortin signaling gene melanocortin 4 receptor (*Mc4r*), compared to WT mice (**Figure 3A**). Agouti-related neuropeptide (*Agrp*), an antagonist of melanocortin receptor signaling, also trended lower in *Mest* ^pKO^ female mice (**Figure 3A**). Hypocretin (*Hcrt*) and the G-protein-coupled receptor hypocretin receptor 1 (*Hcrtr1*), involved in orexigenic signaling and regulation of sleep and arousal, showed no differences in expression between genotypes (**Figure 3A**). Since *Trh* is associated with thyroid-stimulating hormone release and is subject to negative feedback by TH, additional TH-related genes were measured in the hypothalamus (**Figure 3B**). Results showed increased expression of *Dio2* in *Mest* ^pKO^ female mice, which correlated with increased hairless (*Hr*), a recognized TH-responsive gene, possibly indicating a localized TH effect in the hypothalamus (**Figure 3B**). While Kruppel-like factor 9 (*Klf9*), another TH-responsive gene, was marginally elevated in the *Mest* ^pKO^ female hypothalamus, the expression of other recognized TH-responsive genes—uncoupling protein 2 (*Ucp2*), glycerol phosphate dehydrogenase 2 (*Gpd2*), and thyroid hormone responsive (*Thrsp*)—showed no difference in expression between genotypes (**Figure 3B**). *Dio3*, which converts active T3 to inactive metabolites, also showed comparable hypothalamic expression between genotypes (**Figure 3B**). The pituitary showed no differences in gene expression between genotypes (**Figures 3C, D**), whereas the amygdala showed increased expression of *Dio3* in *Mest* ^pKO^ mice, while *Dio2* and *Klf9* were unchanged (**Figure 3E**). The motor cortex showed marginally increased expression of *Dio2* and a small but significant increase in *Klf9* mRNA in *Mest* ^pKO^ female mice, whereas *Dio3* expression was similar for both genotypes (**Figure 3F**). DIO2 and DIO3 enzymatic activity measured in the amygdala and motor cortex correlated with gene expression patterns and showed increased *Dio3* mRNA and DIO3 activity in the amygdala of *Mest* ^pKO^ mice (**Figures 3E–G**). Levels of circulating corticosterone in female WT and *Mest* ^pKO^ mice were comparable (**Supplementary Figure S2**), suggesting that perceived stress is unlikely to be linked with differences in physical activity between genotypes.

Since we previously demonstrated that the conception rate between WT (84.2%) and *Mest* ^pKO^ (82.4%) female mice was comparable (20), we did not assess estrous cyclicity or estrogen levels in this study. In addition, measurements of pituitary expression of luteinizing hormone beta (*Lhb*) and follicle-stimulating hormone beta (*Fshb*)—genes associated with ovulation, estrogen production, follicular development, and control of the estrous cycle—were similar between genotypes (**Supplementary Figure S3**), suggesting normal hormonal patterns.

In contrast to the results in female mice, the expression of the hypothalamic genes *Dio2* and *Hr* in male mice showed no differences between genotypes (**Supplementary Figure S4**). Overall, patterns of gene expression suggested that the increased physical activity of *Mest* ^pKO^ female mice could be at least partially due to increased hypothalamic TH signaling.

A limitation of our study is that circulating thyroid hormones were not measured. However, while not as direct or convincing as measurements of T3 and T4 in serum or plasma, we show that hepatic gene expression of *Dio1* and *Thrsp*—which have been shown to correlate with levels of circulating thyroid hormones (35, 36)—showed no differences between female WT and *Mest* ^pKO^ mice after being fed CD or WD (**Supplementary Figure S5**). These data suggest that localized conversion of T4 to T3 by DIO2 in the hypothalamus contributes to the phenotypic differences in physical activity observed between genotypes.

### 3.4 The hypothalamic transcriptome is altered in control diet-fed female *Mest* ^pKO^ mice

**Figure 4:**
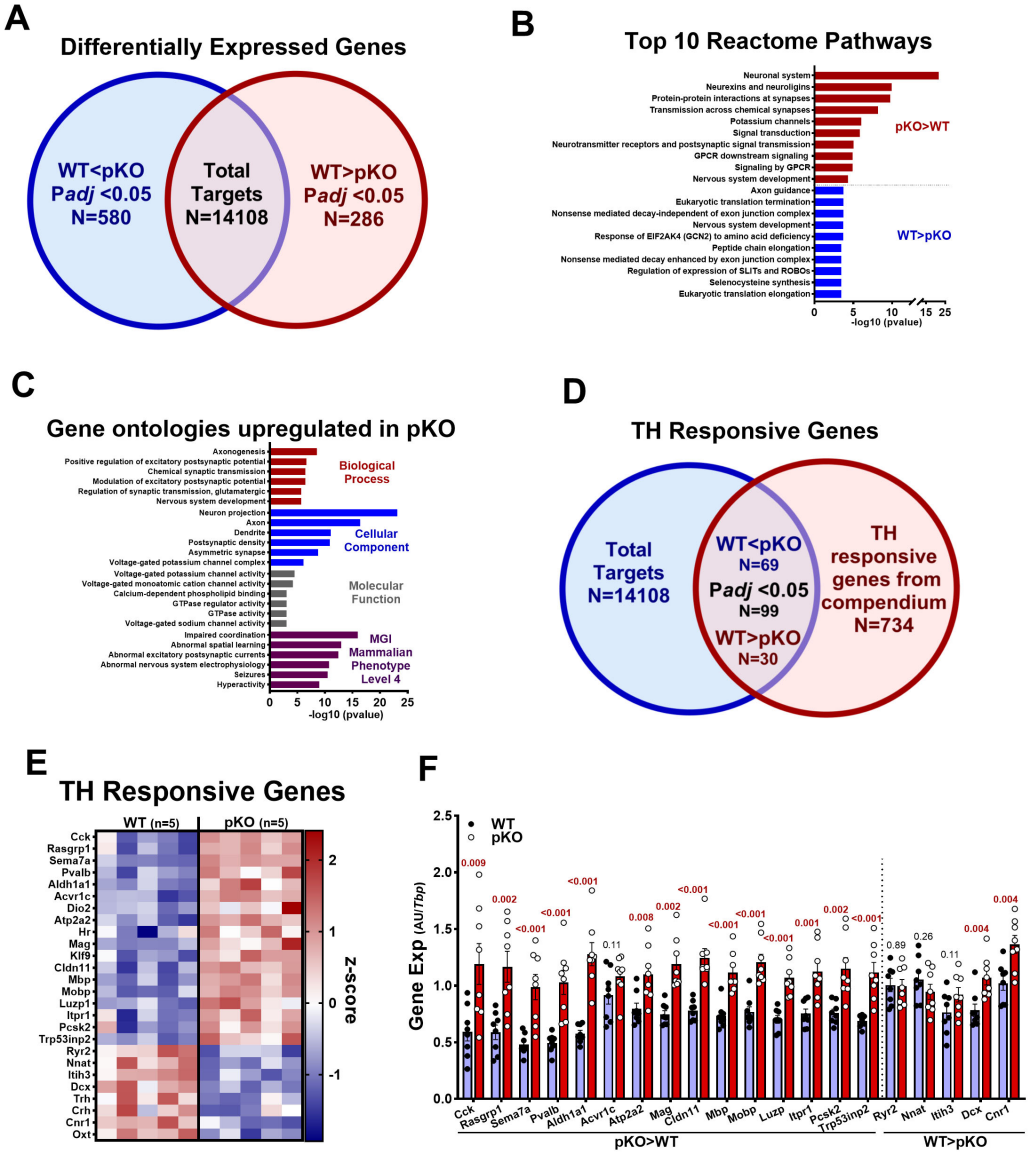
Transcriptomic analyses reveal differential hypothalamic expression patterns between CD-fed WT and *Mest* ^pKO^ (pKO) female mice. **(A)** Venn diagram showing the numbers of differentially expressed genes between WT and *Mest* ^pKO^ female mice and the top 10 Reactome pathways **(B)** associated with genes expressed at higher or lower levels in *Mest* ^pKO^ mice compared to WT. **(C)** Ontology analyses of genes upregulated in the hypothalamus of *Mest* ^pKO^ mice compared to WT. **(D)** Venn diagram showing the overlap between thyroid hormone (TH)-responsive genes from a published compendium (17) and genes differentially expressed between WT and *Mest* ^pKO^ mice. **(E)** Heatmap of TH-responsive genes showing significant differences in expression between WT and *Mest* ^pKO^ mice. **(F)** Differentially expressed TH-responsive genes identified by RNA-seq were validated in hypothalamic RNA from WT (*n* = 9) and *Mest* ^pKO^ (*n* = 9) mice using RT-qPCR. Data in **(F)** were analyzed using unpaired *t*-tests, and *p*-values are numerically indicated with < 0.001 representing a value less than 0.0005.

The RNA-seq transcriptome of WT and *Mest* ^pKO^ female mice, selected from the CD-fed cohort (**Figure 1A**), revealed 866 differentially expressed genes (DEGs; *p*_adj_ < 0.05), with 580 upregulated and 286 downregulated in *Mest* ^pKO^ compared to WT female mice (**Figure 4A**, **Supplementary Table S4**). Functional annotation of upregulated genes showed enrichment in pathways associated with the neuronal system, neurexins and neuroligins, and protein–protein interactions at synapses. Downregulated genes showed enrichment in axon guidance, eukaryotic translation termination, and nonsense-mediated decay independent of the exon junction complex (**Figure 4B**). Gene ontology was consistent with pathway analysis and emphasized enrichment for biological process terms associated with axonogenesis, positive regulation of excitatory postsynaptic potential, and chemical and glutamatergic synaptic transmission, and was consistent with cellular component (neuron projection, axon, dendrite, etc.) and molecular function (voltage-gated potassium and monoatomic cation channel activity) ontologies (**Figure 4C**). MGI Mammalian Phenotype (level 4) analyses showed enrichment for hyperactivity, which closely matches the phenotype observed in *Mest* ^pKO^ female mice (**Figure 4C**).

We next explored the extent of TH involvement in the hypothalamus of CD-fed female mice and identified 99 DEGs (*p*_adj_ ≤ 0.05) from a total of 866 DEGs (**Supplementary Table S4**) that overlapped with a published compendium of 734 top TH-responsive genes (37) (**Figure 4D**), with 69 upregulated and 30 downregulated in *Mest* ^pKO^ compared to WT female mice (**Figures 4D, E**). Fifteen upregulated genes were selected, and their expression was validated by RT-qPCR (**Figure 4F**). While three of the downregulated genes in *Mest* ^pKO^ (*Crh*, *Oxt*, and *Trh*) were validated (**Figure 3A**), an additional five failed to validate by RT-qPCR, with two (*Cnr1*, *Dcx*) showing significantly higher expression in *Mest* ^pKO^ compared to WT mice (**Figure 4F**). Overall, several well-established TH-responsive genes were significantly upregulated in the hypothalamus of female *Mest* ^pKO^ mice, suggesting a role for TH signaling in increased activity-associated behavior. An analysis comparing hypothalamic DEGs in our study with TH-target genes identified in astrocytes and hypothalamus in two additional studies (38, 39) showed an overlap with 21 TH-upregulated and six TH-downregulated genes TH (39), and eight TH-upregulated and eight TH-downregulated genes (38), with *Klf9* and *Hr* being upregulated across all three studies. Hypergeometric analyses (http://nemates.org/MA/progs/overlap_stats.html) of overlap between TH-responsive DEGs from our study identified a representation factor of 2.8 (*p* < 1.3*e*−06) compared to Zekri et al. (39), and 2.4 (*p* < 9.4*e*−04) compared to Wu et al. (38), providing further evidence for a central role of TH in the activity behavior of female *Mest* ^pKO^ mice.

Because loss of *Mest* was thought to mediate mouse behavior via abnormal development of mesodiencephalic dopaminergic neurons, resulting in reduced tyrosine hydrolase (*Th*) protein and dopamine release (22), we investigated DEGs associated with dopamine biosynthesis in our study. Our analyses revealed no downregulated expression of DEGs involved in dopamine biosynthesis in the hypothalamus of female *Mest* ^pKO^ mice; however, three DEGs—*Th* (*p*_adj_ < 0.001; 1.5F), *Slc6a3* (*p*_adj_ = 0.0017; 5.6F), and *Gpr37* (*p*_adj_ = 0.0037; 1.3F)—showed significant upregulation of expression compared to female WT mice. Gene ontology analyses of DEGS with elevated expression in *Mest* ^pKO^ did not identify significant pathways associated with dopamine synthesis or function. Similarly, pathways associated with catecholamine secretion, transport, uptake, and biosynthesis showed no significant differences between genotypes. Our results using *Mest* ^tm1.2RKz^ are contradictory to past studies using *Mest* knockout mice (e.g., *Mest* ^tm1Masu^), which could be due to differences in the design of the targeted allele (20, 21).

Loss of *Mest*, a paternally expressed imprinted gene, is associated with differential expression of other imprinted genes, including *Klf14*, a maternally expressed gene (MEG), and several paternally expressed genes (PEGs; *Peg3*, *Ndn*, *Dlk1*, *Magel2*; **Supplementary Figure S6**). It is possible that *Mest* disruption acts *in trans* on the expression of other MEGs or PEGs as a component of a network of imprinted genes (40).

### 3.5 Increased adipose expression of *Dio2* and *Ucp1* in female *Mest* ^pKO^ mice fed WD

**Figure 5:**
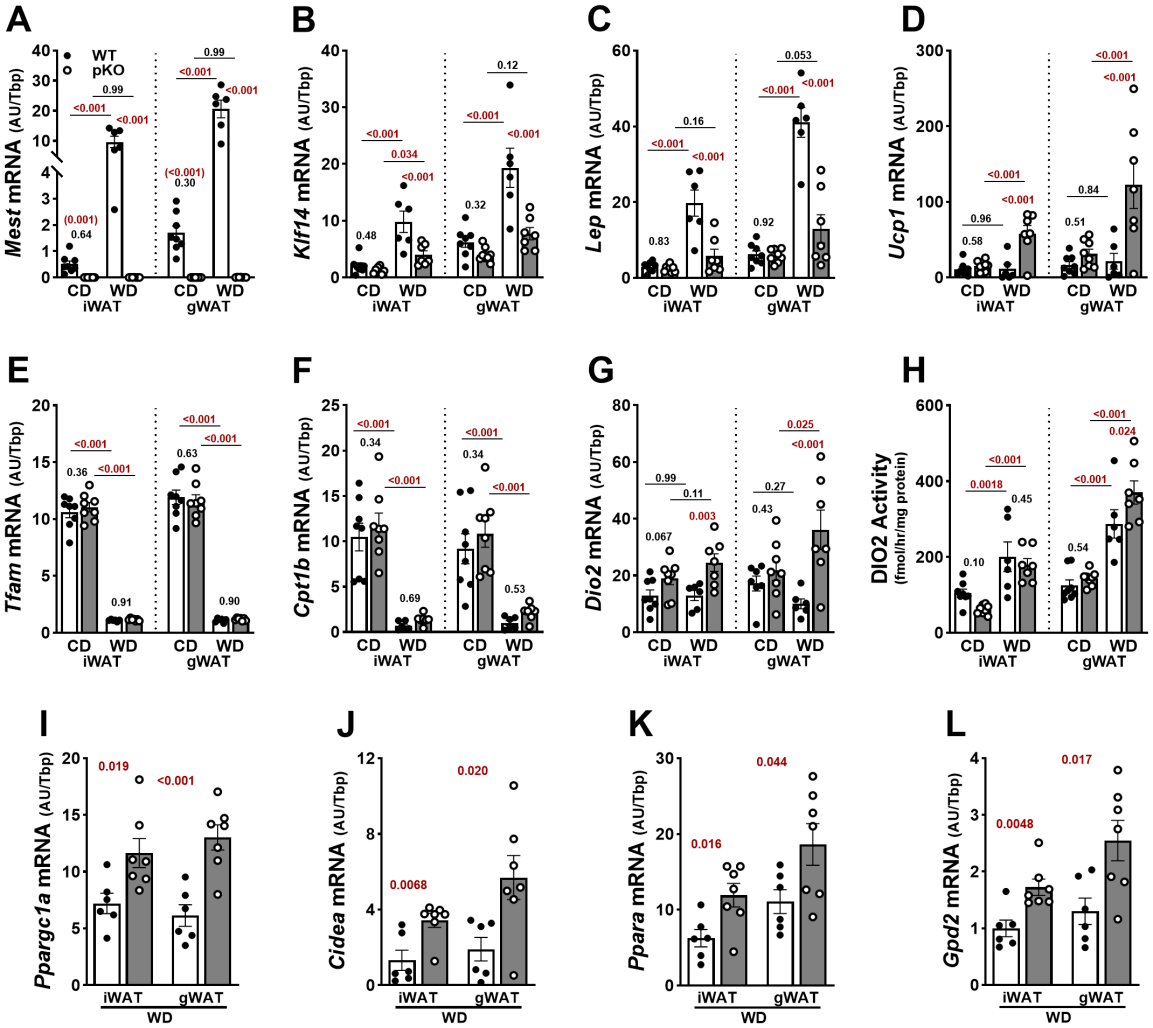
Increased adipose tissue expression of *Ucp1* and *Dio2* in female *Mest* ^pKO^ (pKO) mice fed a Western diet. **(A)** RT-qPCR measurements of gene expression in inguinal (iWAT) and gonadal (gWAT) white adipose tissue show inactivation of *Mest* in female *Mest* ^pKO^ mice. **(B**, **C)** Genes associated with fat mass expansion showed reduced expression in both WAT depots of *Mest* ^pKO^ mice fed WD compared with WT. **(D)** While the thermogenic gene *Ucp1* showed increased expression in *Mest* ^pKO^ mice when fed WD compared to WT, genes associated with **(E)** mitochondrial gene transcription and **(F)** fatty acid transport and oxidation showed no differences between genotypes, but expression was reduced in WD-fed mice. **(G)** *Dio2* expression and **(H)** enzymatic activity were higher in gWAT of *Mest* ^pKO^ mice fed WD compared with WT. **(I**–**L)** Additional genes associated with adipose thermogenesis and glycerol-3-phosphate cycling measured in iWAT and gWAT showed increased expression in *Mest* ^pKO^ mice when fed WD. Data in **(A–H)** were analyzed using two-way ANOVA, and in **(I**–**L)** by unpaired *t*-tests. *p*-values are numerically indicated, and data annotated with < 0.001 indicate a *p*-value of less than 0.0005.

We previously determined that upregulation of *Mest* in iWAT and gWAT in WT female and male mice fed a high-fat diet shows coordinated expression patterns with Krüpple-like factor 14 (*Klf14*), a maternally expressed gene ~ 230 kb downstream of *Mest* on mouse *Chr* 6 (41, 42). Herein, similar coregulation between *Mest* and *Klf14* was observed in WAT depots of female WT mice fed CD (iWAT, *R* ^2^ = 0.75; gWAT, *R* ^2^ = 0.64) and WD (iWAT, *R* ^2^ = 0.82; gWAT, *R* ^2^ = 0.81), and *Mest* was absent in the WAT of female *Mest* ^pKO^ mice (**Figures 5A, B**). *Lep* showed comparable expression between genotypes in iWAT and gWAT in mice fed CD, but its expression was significantly lower in WAT depots of *Mest* ^pKO^ mice compared to WT when fed WD, consistent with reduced adipose mass between genotypes (**Figure 5C**). While *Ucp1* was comparable between genotypes when fed CD, WD-fed *Mest* ^pKO^ female mice showed significantly elevated *Ucp1* in both WAT depots compared to WT mice (**Figure 5D**). In contrast, *Tfam*, a mitochondrial transcription factor, and *Cpt1b*, which plays a role in mitochondrial fatty acid oxidation, were significantly downregulated in both WAT depots of mice fed WD compared to CD, with no differences observed between genotypes (**Supplementary Figures S5E, F**). *Dio2* showed similar patterns of expression as *Ucp1* and was upregulated in both iWAT and gWAT of *Mest* ^pKO^ mice fed WD (**Figure 5G**). Measurements of DIO2 activity in WAT depots corresponded poorly with gene expression but showed increased activity in both WAT depots in mice fed WD, with significantly higher levels in gWAT of *Mest* ^pKO^ mice compared with controls (**Figure 5H**). Additional screening of thermogenesis-associated genes in WAT (*Ppargc1a*, *Cidea*, *Ppara*, and *Gpd2*) showed expression patterns consistent with *Ucp1* in WAT depots of WD-fed *Mest* ^pKO^ mice (**Figures 5I–L**), which were strongly influenced by genotype (**Supplementary Table S5**). These data are further supported by increased “beiging” of iWAT and gWAT in histological sections of WD-fed *Mest* ^pKO^ female mice compared with WT (**Supplementary Figure S7**), suggestive of increased UCP1 thermogenesis and EE. In total, our data suggest that increased WAT thermogenesis and SPA likely contribute to the reduced susceptibility of *Mest* ^pKO^ female mice to dietary obesity.

## 4 Discussion

Previous studies in our laboratory and others show *Mest* is predominantly expressed from the paternal allele in WAT (12, 43, 44), with concomitant levels of WAT *Mest* mRNA and MEST protein correlating with dietary fat-induced FM accretion (8, 9, 41), and reduced diet-induced obesity in mice with global or adipocyte-specific inactivation of the paternal allele of *Mest* (12). While our earlier studies focused on the role of *Mest* in male B6 mice, we recently determined that female B6 mice show dietary-fat inducible *Mest* in WAT (~ 27-fold), which is comparable to male mice (~ 24-fold), albeit with lower baseline expression levels (42). Therefore, dietary fat-induced *Mest* expression in WAT is a phenomenon common to both sexes.

Herein, we focused on understanding the metabolic phenotypes of female mice with paternal inactivation of *Mest* under two dietary conditions. *Mest* ^pKO^ female mice fed CD were significantly leaner than WT, with reduced FM and LM, which was further accentuated after feeding WD. Protection of *Mest* ^pKO^ female mice from dietary obesity was associated with improved glucose tolerance and insulin sensitivity. *Mest* ^pKO^ female mice fed CD show noteworthy adaptations in energy metabolism, including increased EE and a trend toward increased locomotor activity compared to WT, enabling maintenance of a lean phenotype over time. Remarkably, when fed WD, *Mest* ^pKO^ female mice responded to increased caloric intake by substantially increasing locomotor activity and SPA, translating to increased NEAT compared to WT female mice. In addition, *Mest* ^pKO^ female mice allocated more time moving at increased speed, engaged in fine movements, and spent less time staying still and sleeping compared to WT when fed WD.

A notable phenotype of *Mest* ^pKO^ female mice, the interaction of diet and SPA, highlights an important strategy to counteract dietary obesity. Increased physical activity of *Mest* ^pKO^ female mice corresponded to fine movements such as walking, grooming, and scratching that are not associated with exercise. This was reflected in significantly elevated values for NEAT and fine movements in WD-fed *Mest* ^pKO^ female mice compared to WT (**Figures 2N, O**). In human terms, this corresponds to the energy utilized to walk, perform housework, undertake agricultural tasks, and fidget (45). Fidgeting and other small movements are spontaneous and random, triggering body and limb movements and locomotion (46). In industrialized societies, human locomotion, essential for daily activities, is low compared to that of our ancestors (47). However, harnessing this “fidget” factor in humans has proven beneficial in resisting fat gain caused by increased caloric intake (46, 48). Individuals who gained less weight under caloric excess were observed to have increased EE associated with nonexercise movements, which was equivalent to up to 700 kcal/day above usual EE (17).

The specifics of neural control of SPA and NEAT are still being resolved. In contrast to exercise, which is governed by higher-level cortical function, SPA is likely facilitated by more primitive areas of the brain, such as the hypothalamus (16). Neuromediators implicated in SPA, and consequently NEAT, include hypocretin/orexin, *Agrp*, ghrelin (*Ghrl*), neuromedin U (*Nmu*), *Crh*, cholecystokinin (*Cck*), estrogen, leptin, and dopamine (16, 19, 49), some of which exhibit altered expression in the hypothalamus of *Mest* ^pKO^ female mice. Furthermore, studies have demonstrated that rats supplemented with T3 show increased SPA and NEAT (50).

Thyroid hormones are well recognized for their profound effects on energy expenditure and body weight regulation. The changes mediated by thyroid hormones in energy expenditure require a signal from the brain, which alters local hypothalamic *Dio2* expression, with consequent changes in the regulation of energy balance (51). The enhanced SPA phenotype in WD-fed *Mest* ^pKO^ female mice presents a model to investigate neural mechanisms of NEAT in relation to TH signaling and energy metabolism. TH controls an array of developmental and physiological processes in the brain (37). We observe upregulation of *Dio2* in the *Mest* ^pKO^ female hypothalamus, which suggests a possible TH-regulated mechanism in the control of physical activity. TH that reaches the brain through the circulation undergoes tightly controlled metabolism, leading to hormonal activation or inactivation. *Dio2* can locally increase TH signaling by converting the inactive prohormone T4 into the biologically active T3 in a tissue- and temporal-specific fashion, independent of circulating hormone levels (52). *Dio2-*generated T3 from glial cells can influence neighboring neural cell types, acting in a paracrine fashion to modulate T3-responsive genes (53). In humans, TH dysfunction is linked to sedentary behavior in hypothyroidism, as opposed to increased activity in hyperthyroidism (54). Pharmacologic mouse models of hypo- and hyperthyroidism recapitulate human phenotypes, including alterations in overall physical activity (55). Interestingly, male mice with astrocyte-specific *Dio2* inactivation have normal serum T3 but exhibit anxiety-depressive behavior linked to decreased hippocampal expression of classic TH-responsive genes (56). The increased hypothalamic *Dio2* expression and the large number of TH-responsive genes upregulated in the hypothalamus of the *Mest* ^pKO^ female mice suggest a local increase in T3 levels, which may augment SPA. In addition, peripheral tissue T3 effects elicited by *Dio2* upregulation in *Mest* ^pKO^ female mice are demonstrated by increased WAT *Ucp1*, a recognized T3-responsive gene (57). *Mest* ^pKO^ female mice fed WD showed markedly increased *Dio2* and *Ucp1* in WAT, in addition to genes associated with thermogenesis and fatty acid oxidation. Although a mechanism for the functional association between MEST and DIO2 remains to be resolved, it is of marked interest that both are colocalized subcellularly in the endoplasmic reticulum membrane (13, 58), unlike DIO1 and DIO3, which are found in the plasma membrane (58). This further supports the possibility that disruption of *Mest* leads to DIO2-dependent local activation of TH signaling in the brain and WAT.

Imprinted genes are highly expressed in the brain and influence synaptic function and plasticity, neural development and wiring, social behaviors, energy balance, and cognition, among other processes (59, 60). In the adult mouse brain, MEST has been reported in neuron-rich areas by *lac*Z staining (21), and more recent advanced technologies have described its expression in brain regions such as the pituitary (61), hypothalamus (62–64), and mouse forebrain (65). Single-cell RNA sequencing data indicate that *Mest* is expressed in neurons and nonneuronal populations, e.g., astrocytes and tanycytes (64), and it was shown to be expressed in neurons and tanycytes of female mice regardless of age (63). *Mest* is also highly expressed in the lateral ganglionic eminence of the mouse forebrain, which gives rise to all forebrain GABAergic cells (65), is among the top 1% of enriched imprinted genes in the embryonic and adult mouse pituitary, and scRNA sequencing showed high *Mest* expression in lactotrophs and thyrotrophs in the postnatal d4 and d49 anterior pituitary (61). The unique increased physical activity phenotype observed in *Mest* mutant female mice further supports the essential role of imprinted genes in the brain.

Since male mice with adipocyte-specific inactivation of *Mest* show similar resistance to diet-induced obesity as mice with global inactivation of *Mest* (12), it is possible that metabolic imbalance caused by the reduced capacity for lipid storage in adipocytes in the absence of *Mest* in female mice may drive central nervous system regulation of SPA. Alternatively, reduced dietary fat-induced obesity in female mice with global inactivation of *Mest* could result from an additive contribution from both adipose tissue and the central nervous system (CNS), or from a sex-specific CNS-dominant mechanism. Future investigation of a probable mechanistic link between *Mest* and TH metabolism in the hypothalamus will determine the effects of adipocyte- or neural cell-specific inactivation of *Mest*, distinguishing between peripheral and central effects of *Mest* inactivation on metabolism and SPA in female mice. Moreover, differences in physical activity between male and female *Mest* ^pKO^ mice are not well understood but may involve developmental programming of the neonatal brain by reproductive hormones such as estrogen, which is recognized for motivating behavioral changes in female mice (66).

## Data Availability

The datasets presented in this study can be found in online repositories. The names of the repository/repositories and accession number(s) can be found in the article/**Supplementary Material**.

## Supplementary material

Supplementary Figure 1 Phenotypic analyses of WT and *Mest* ^pKO^ (pKO) male mice fed control (CD) and western diet (WD). **(A)** BWs of WT and *Mest* ^pKO^ male mice fed CD and **(B)** WD from 8 to 16 weeks of age. **(C)** Fat mass, **(D)** % fat mass, **(E)** lean mass and **(F)** % lean mass of CD fed male WT and *Mest* ^pKO^ mice at 8, 12 and 16 weeks of age measured by NMR. **(G)** Fat mass, **(H)** % fat mass, **(I)** lean mass and **(J)** % lean mass of WD fed male mice at 8, 12 and 16 weeks of age. All data were analyzed by two-way ANOVA. Annotation with a, b, c, or d indicate p-values lower than 0.05, 0.01, 0.001 and 0.0001 for data in figure panels **(A, B)** and indicated numerically in figure panels **(C-J)**.

Supplementary Figure 2 Serum corticosterone levels are similar between genotypes when fed either control (CD) or Western diet (WD). Serum corticosterone was measured in WT and *Mest* ^pKO^ (pKO) mice fed CD (n=7 per genotype) or WD (n=15–17 per genotype). Unpaired t-tests were used to measure significance between genotypes for each diet.

Supplementary Figure 3 Pituitary expression of *Lhb* and *Fshb* is comparable in the pituitary of female WT and *Mest* ^pKO^ (pKO) mice fed CD. Gene expression measured in RNA from pituitary of WT (n=7) and *Mest* ^pKO^ (n=7) mice. Unpaired t-tests were used to measure significance between genotypes for each diet. P-values are numerically indicated.

Supplementary Figure 4 Hypothalamic expression of metabolic and thyroid-hormone responsive genes is similar between male WT and *Mest* ^pKO^ (pKO) mice fed WD. Gene expression measured in RNA isolated from hypothalamus of WT (n=8) and *Mest* ^pKO^ (n=7) mice showed no significant differences in thyroid metabolic (*Dio2*, *Dio3*), regulatory (*Crh*) or responsive (*Hr*, *Klf9*, *Mbp*) genes between genotypes. *Mest* mRNA is mostly absent in hypothalamus of *Mest* ^pKO^ mice. Unpaired t-tests were used to measure significance between genotypes. P-values are numerically indicated and data annotated with <0.001 indicates a p-value of less than 0.0005.

Supplementary Figure 5 Hepatic thyroid hormone responsive genes show similar expression in female WT and *Mest* ^pKO^ (pKO) mice fed CD or WD. *Dio1* and *Thrsp* gene expression was measured in RNA isolated from liver of WT (n=7-8) and *Mest* ^pKO^ (n=7-8) mice. Unpaired t-tests were used to measure significance between genotypes for each diet. P-values are numerically indicated.

Supplementary Figure 6 Hypothalamic imprinted genes are differentially expressed between female WT and *Mest* ^pKO^ (pKO) mice fed CD. Gene expression measured in RNA from hypothalamus of WT (n=8) and *Mest* ^pKO^ (n=8) mice. The letters in parentheses below each gene indicate the imprinting status as maternal-expressed (m) or paternal-expressed (p). Unpaired t-tests were used to measure significance between genotypes for each diet. P-values are numerically indicated.

Supplementary Figure 7 Adipocyte morphology of gonadal (gWAT) and inguinal (iWAT white adipose tissue (WAT) of wildtype (WT; n=4) and *Mest* **^pKO^** (pKO; n=4) female mice after being fed Western diet (WD) for 4 weeks. Data shows smaller adipocytes in both WAT depots in WD-fed female *Mest* **^pKO^** mice compared to WT.
