# Supplementary figures and images for: Increased spontaneous physical activity in female MEST-deficient mice protects against diet-induced obesity
Source: Front Endocrinol (Lausanne). 2025 Oct 29;16:1680158. doi: 10.3389/fendo.2025.1680158 (PMC12609188; doi:10.3389/fendo.2025.1680158)

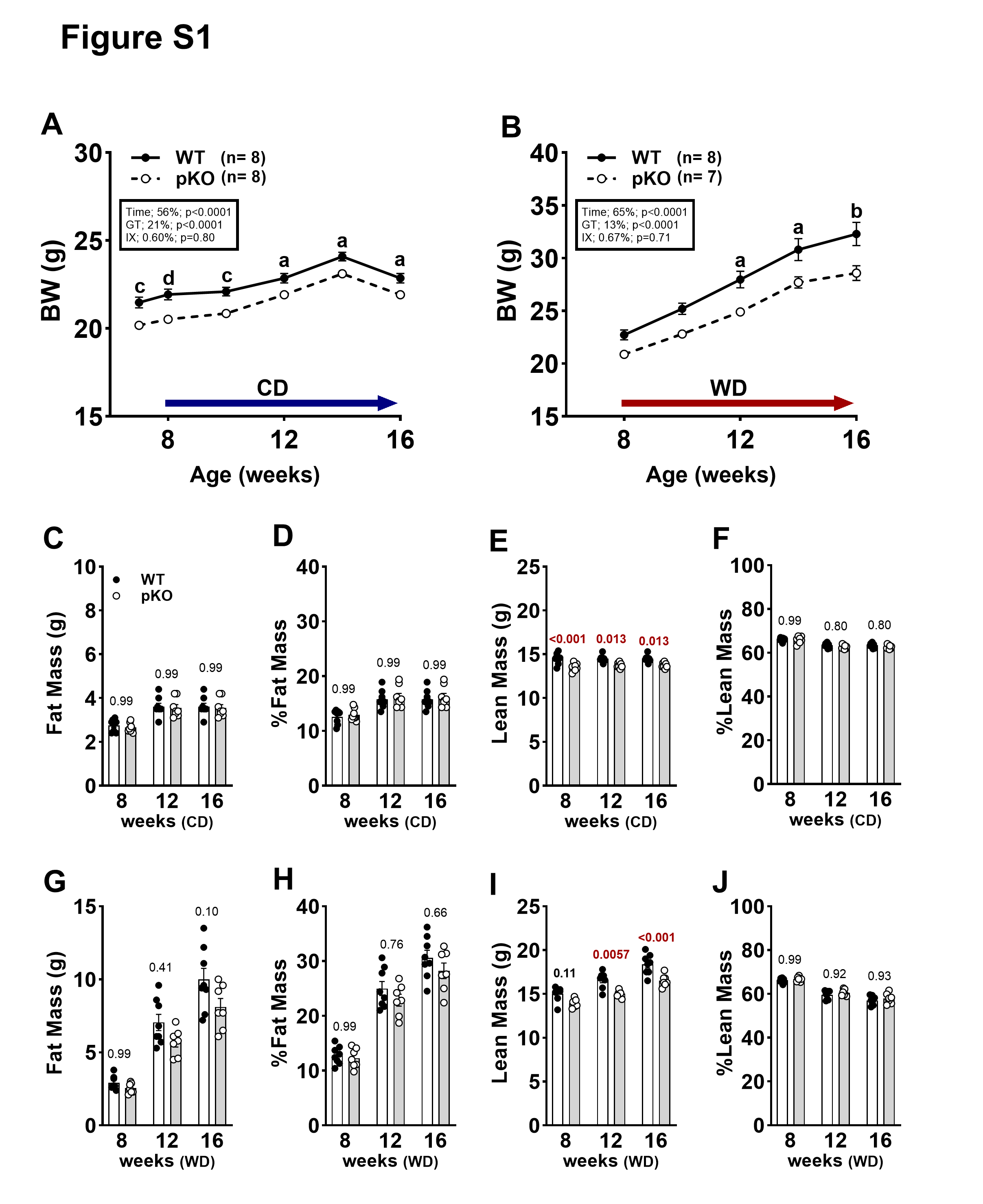

Supplement: Supplementary Figure 1 — Phenotypic analyses of WT and Mest pKO (pKO) male mice fed control (CD) and western diet (WD). (A) BWs of WT and Mest pKO male mice fed CD and (B) WD from 8 to 16 weeks of age. (C) Fat mass, (D) % fat mass, (E) lean mass and (F) % lean mass of CD fed male WT and Mest pKO mice at 8, 12 and 16 weeks of age measured by NMR. (G) Fat mass, (H) % fat mass, (I) lean mass and (J) % lean mass of WD fed male mice at 8, 12 and 16 weeks of age. All data were analyzed by two-way ANOVA. Annotation with a, b, c, or d indicate p-values lower than 0.05, 0.01, 0.001 and 0.0001 for data in figure panels (A, B) and indicated numerically in figure panels (C-J). [file Image1.tif]

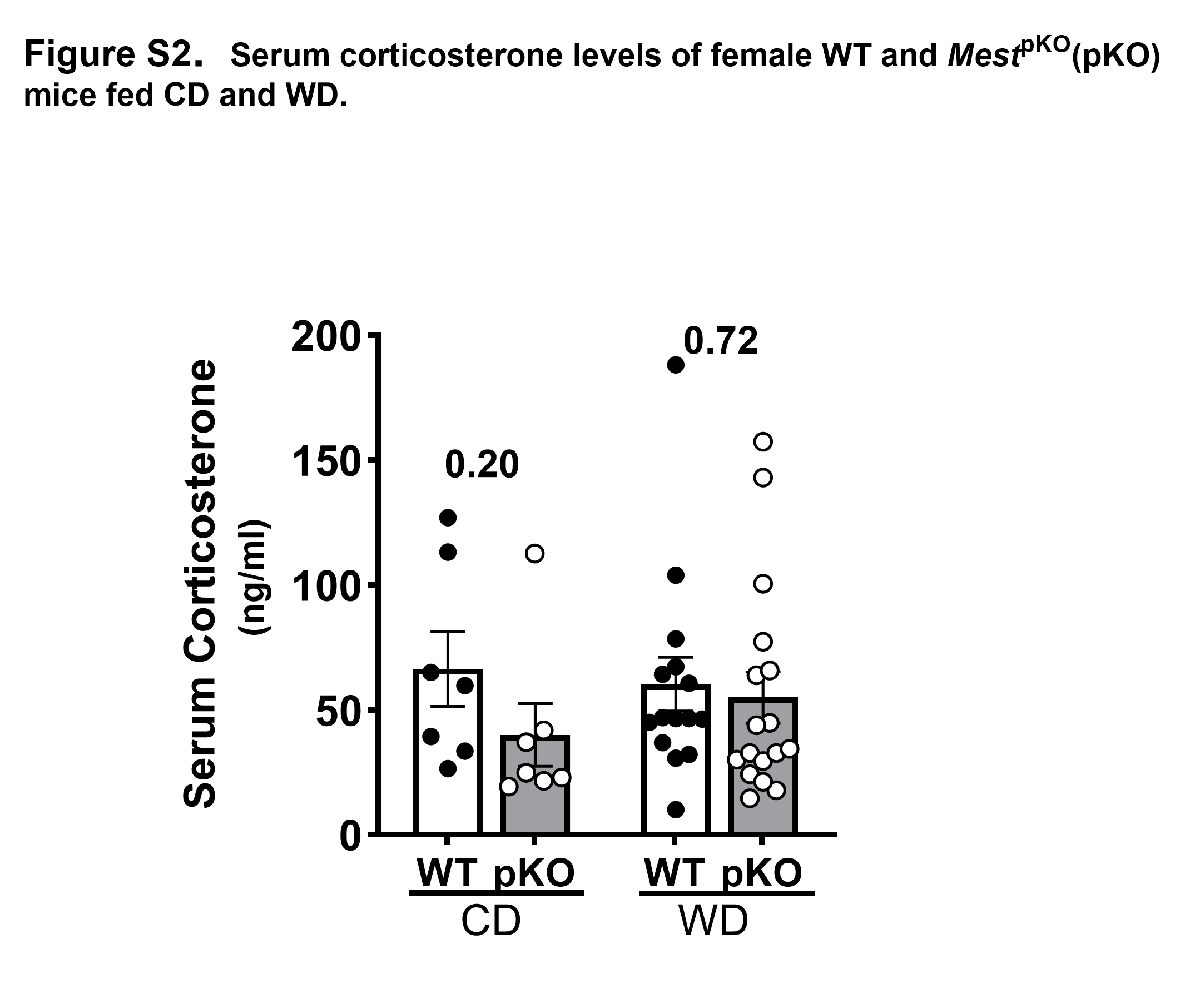

Supplement: Supplementary Figure 2 — Serum corticosterone levels are similar between genotypes when fed either control (CD) or Western diet (WD). Serum corticosterone was measured in WT and Mest pKO (pKO) mice fed CD (n=7 per genotype) or WD (n=15–17 per genotype). Unpaired t-tests were used to measure significance between genotypes for each diet. [file Image2.tif]

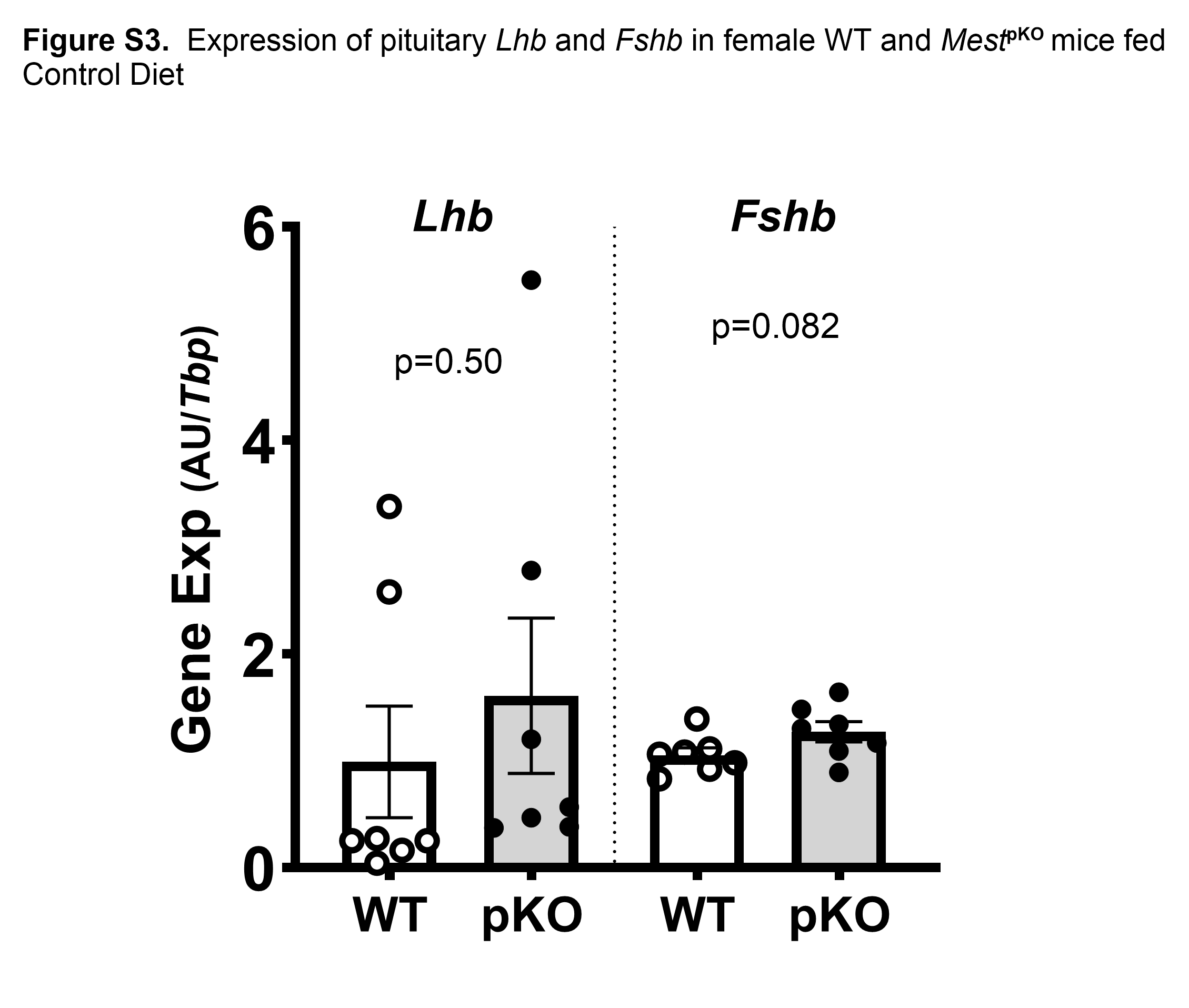

Supplement: Supplementary Figure 3 — Pituitary expression of Lhb and Fshb is comparable in the pituitary of female WT and Mest pKO (pKO) mice fed CD. Gene expression measured in RNA from pituitary of WT (n=7) and Mest pKO (n=7) mice. Unpaired t-tests were used to measure significance between genotypes for each diet. P-values are numerically indicated. [file Image3.tif]

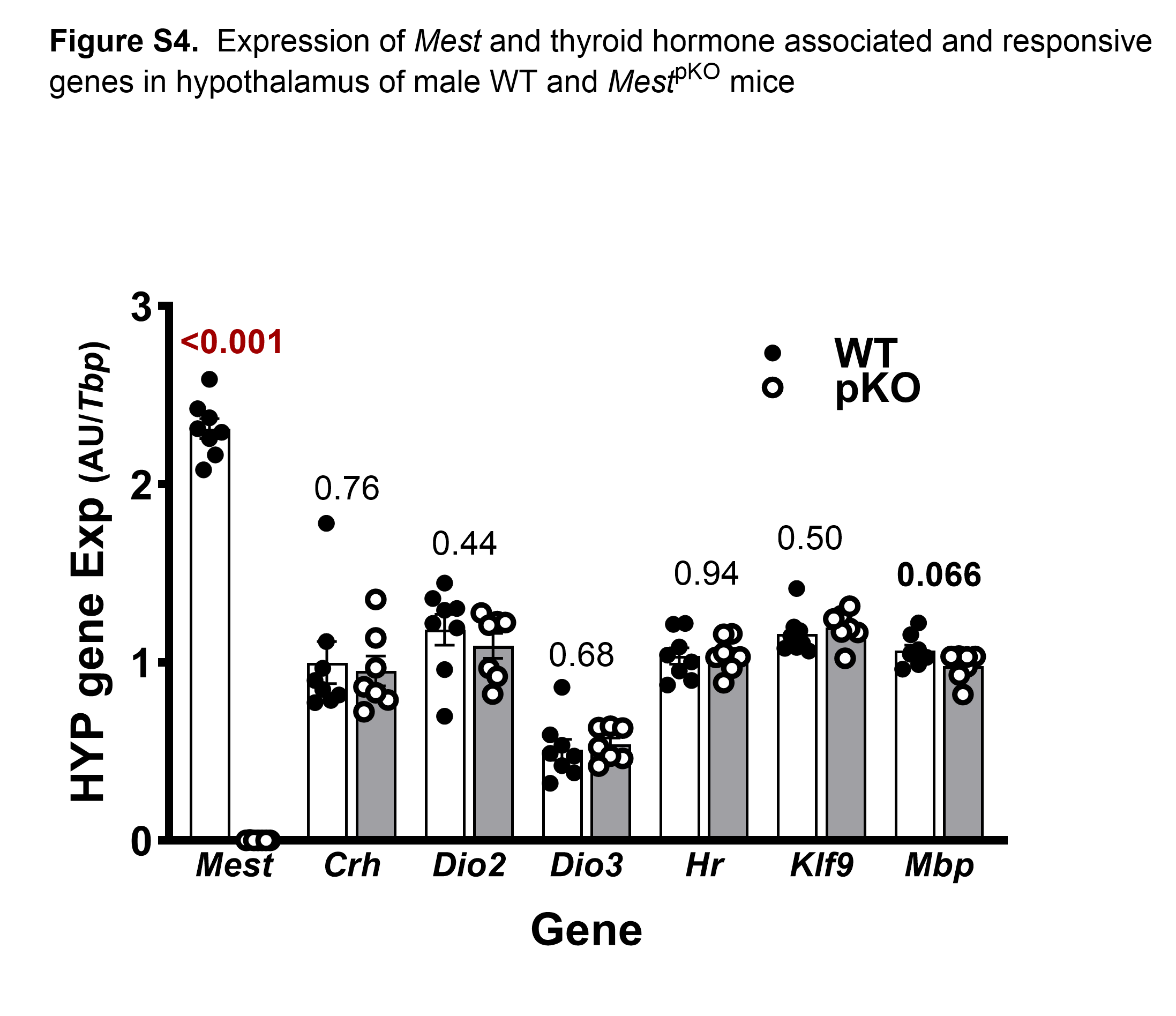

Supplement: Supplementary Figure 4 — Hypothalamic expression of metabolic and thyroid-hormone responsive genes is similar between male WT and Mest pKO (pKO) mice fed WD. Gene expression measured in RNA isolated from hypothalamus of WT (n=8) and Mest pKO (n=7) mice showed no significant differences in thyroid metabolic (Dio2, Dio3), regulatory (Crh) or responsive (Hr, Klf9, Mbp) genes between genotypes. Mest mRNA is mostly absent in hypothalamus of Mest pKO mice. Unpaired t-tests were used to measure significance between genotypes. P-values are numerically indicated and data annotated with <0.001 indicates a p-value of less than 0.0005. [file Image4.tif]

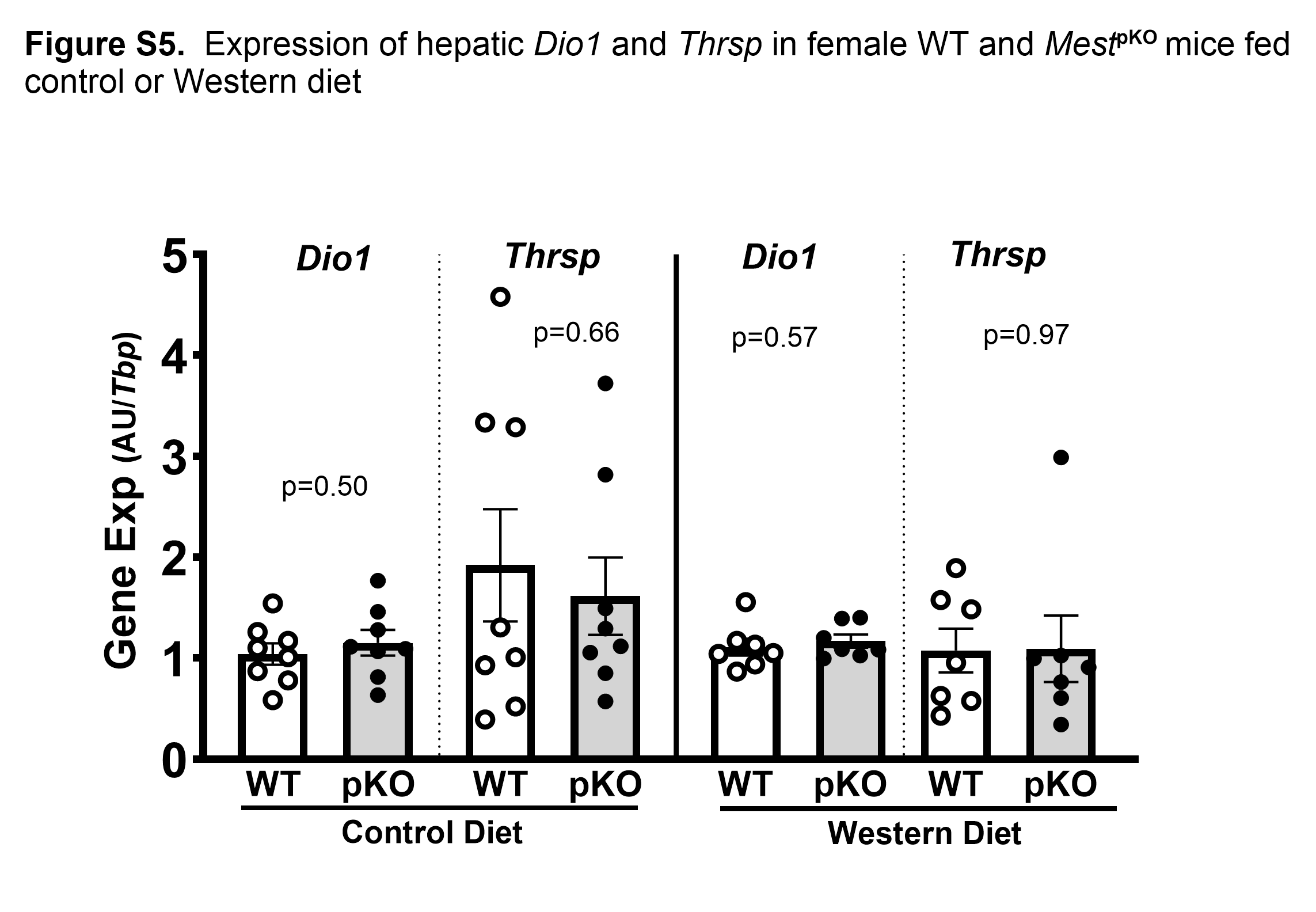

Supplement: Supplementary Figure 5 — Hepatic thyroid hormone responsive genes show similar expression in female WT and Mest pKO (pKO) mice fed CD or WD. Dio1 and Thrsp gene expression was measured in RNA isolated from liver of WT (n=7-8) and Mest pKO (n=7-8) mice. Unpaired t-tests were used to measure significance between genotypes for each diet. P-values are numerically indicated. [file Image5.tif]

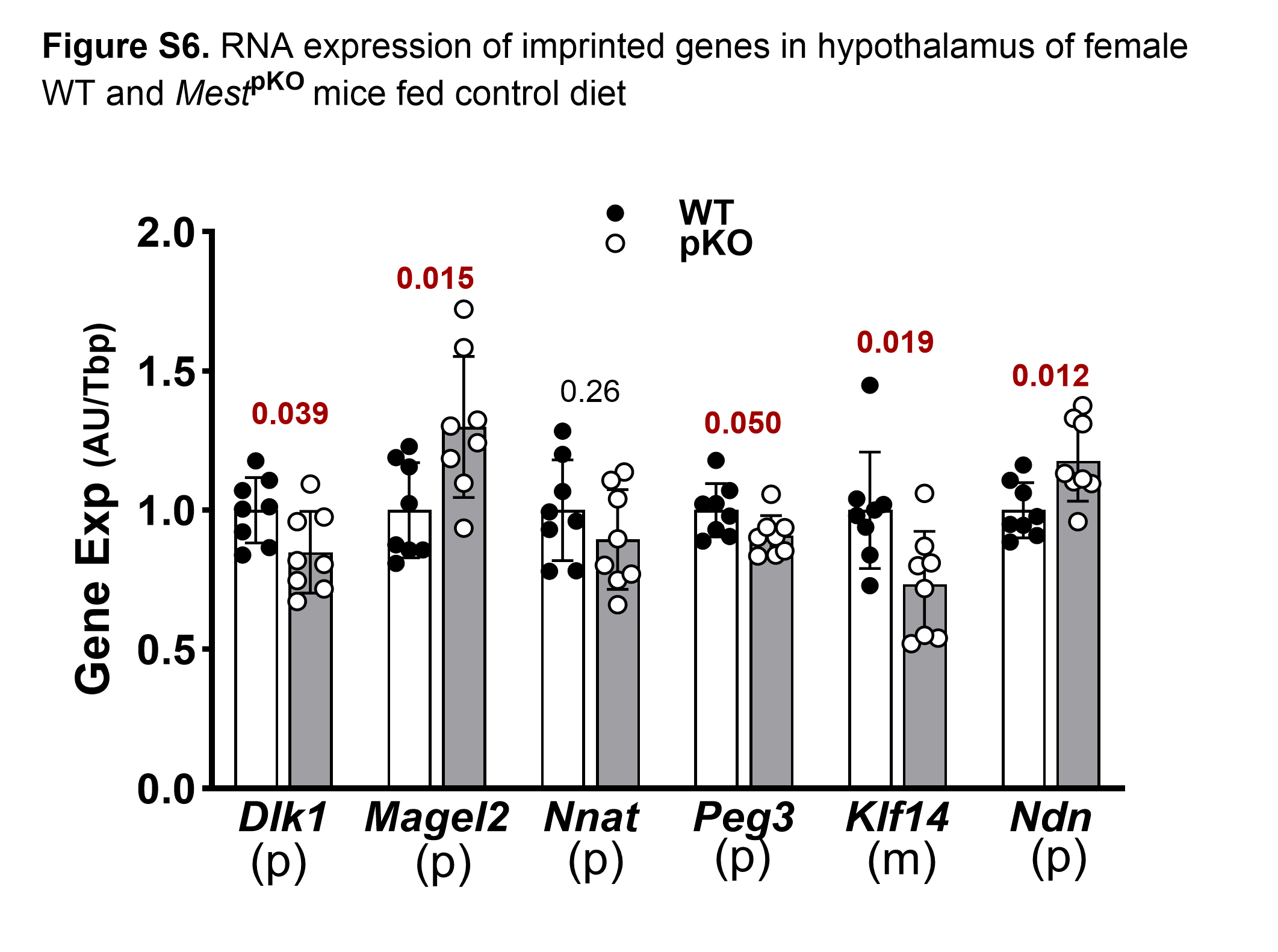

Supplement: Supplementary Figure 6 — Hypothalamic imprinted genes are differentially expressed between female WT and Mest pKO (pKO) mice fed CD. Gene expression measured in RNA from hypothalamus of WT (n=8) and Mest pKO (n=8) mice. The letters in parentheses below each gene indicate the imprinting status as maternal-expressed (m) or paternal-expressed (p). Unpaired t-tests were used to measure significance between genotypes for each diet. P-values are numerically indicated. [file Image6.tif]

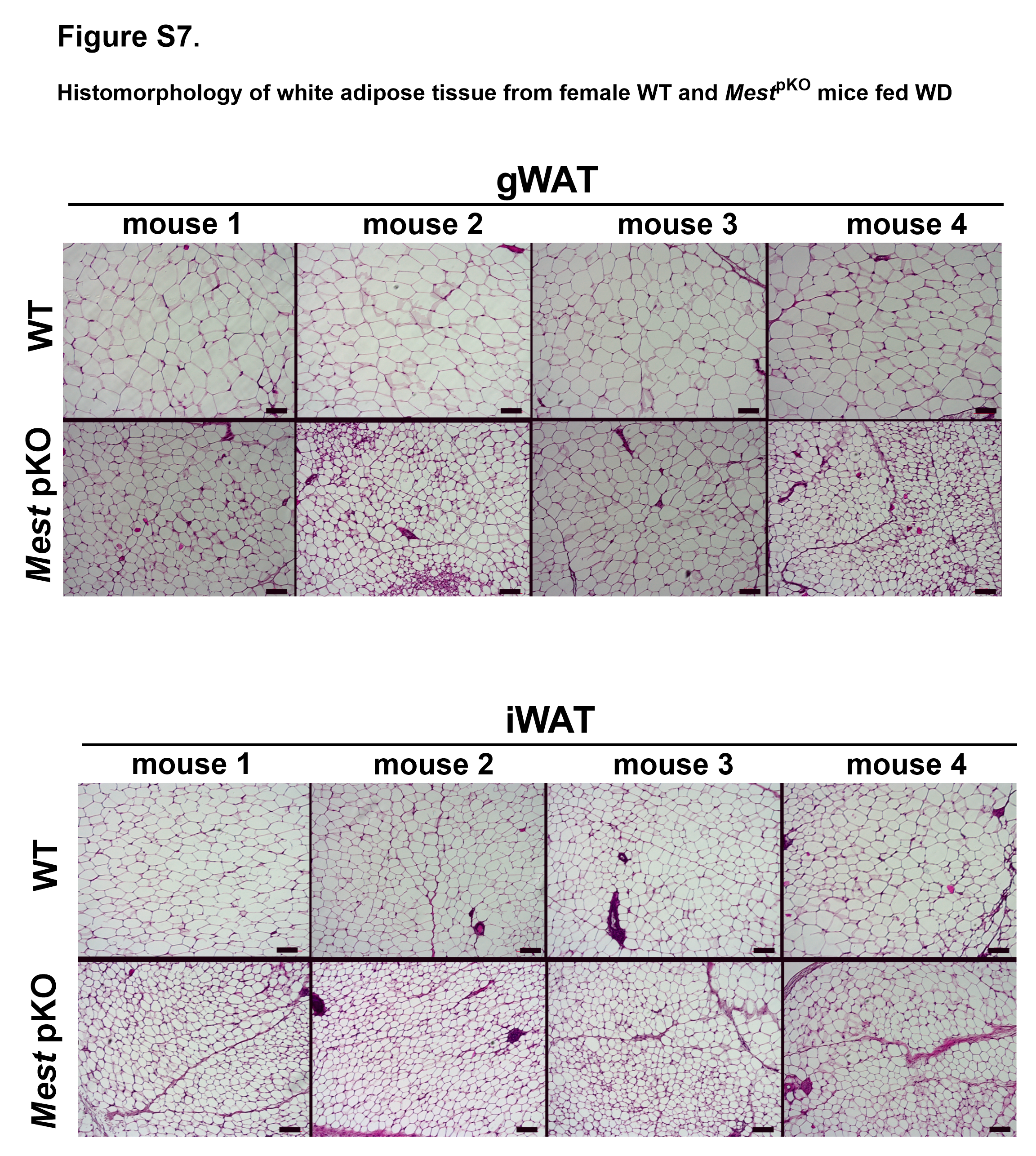

Supplement: Supplementary Figure 7 — Adipocyte morphology of gonadal (gWAT) and inguinal (iWAT white adipose tissue (WAT) of wildtype (WT; n=4) and Mest pKO (pKO; n=4) female mice after being fed Western diet (WD) for 4 weeks. Data shows smaller adipocytes in both WAT depots in WD-fed female Mest pKO mice compared to WT. [file Image7.tif]
