# Supplementary material for: Increased spontaneous physical activity in female MEST-deficient mice protects against diet-induced obesity
Source: Front Endocrinol (Lausanne). 2025 Oct 29;16:1680158. doi: 10.3389/fendo.2025.1680158 (PMC12609188; doi:10.3389/fendo.2025.1680158)
Supplement: Supplementary file 8 [file Table1.docx]

**Table S1**

**Panel A**

**Body weight and composition analyses (2W-ANOVA) of female mice fed CD or WD**

|  |  |  |  |  | **TIME (wks)** | | **GENOTYPE** | | **INTERACTION** | |
| --- | --- | --- | --- | --- | --- | --- | --- | --- | --- | --- |
| **Diet** | **Sex** | **WT (n)** | ***Mest*^pko^ (n)** | **Phenotype** | **% var** | **pvalue** | **% var** | **pvalue** | **% var** | **pvalue** |
| CD | F | 8-9 | 8-11 | BW (g) | 45.8 | **<0.0001** | 14.2 | **<0.0001** | 0.71 | 0.97 |
| CD | F | 8-9 | 8-11 | Fat Mass (g) | 51.3 | **<0.0001** | 3.04 | 0.072 | 2.25 | 0.30 |
| CD | F | 8-9 | 8-11 | % Fat Mass | 63.1 | **<0.0001** | 0.32 | 0.51 | 1.30 | 0.41 |
| CD | F | 8-9 | 8-11 | Lean Mass (g) | 60.4 | **<0.0001** | 6.00 | **0.0035** | 0.15 | 0.89 |
| CD | F | 8-9 | 8-11 | % Lean Mass | 47.1 | **<0.0001** | 0.38 | 0.54 | 3.81 | 0.15 |
| WD | F | 6 | 7 | BW (g) | 45.5 | **<0.0001** | 35.9 | **<0.0001** | 4.62 | **0.0006** |
| WD | F | 6 | 7 | Fat Mass (g) | 36.4 | **<0.0001** | 35.0 | **<0.0001** | 11.6 | **0.0005** |
| WD | F | 6 | 7 | % Fat Mass | 32.5 | **<0.0001** | 36.3 | **<0.0001** | 9.00 | **0.0059** |
| WD | F | 6 | 7 | Lean Mass (g) | 59.2 | **<0.0001** | 25.3 | **<0.0001** | 2.28 | 0.091 |
| WD | F | 6 | 7 | % Lean Mass | 9.89 | **0.012** | 46.5 | **<0.0001** | 13.0 | **0.0037** |

**Panel B**

**Glucose tolerance test of female mice fed CD or WD**

| **Control Diet** | **Sex** | **Genotype** | **N** | **0 min** | **30 min** | **60 min** | **90 min** | **120 min** |
| --- | --- | --- | --- | --- | --- | --- | --- | --- |
|  | Female | WT | 8 | 80.8 ± 4.8 | 298 ± 34 | 183 ± 29 | 129 ± 14 | 118 ± 16 |
|  | Female | *Mest*^pKO^ | 8 | 76.0 ± 6.9 | 346 ± 46 | 231 ± 36 | 140 ± 10 | 120 ± 7.0 |
| **Pvalue=** |  |  |  | 0.13 | **0.032** | **0.011** | 0.096 | 0.75 |
|  |  |  |  |  |  |  |  |  |
| **Western Diet** | **Sex** | **Genotype** | **N** | **0 min** | **30 min** | **60 min** | **90 min** | **120 min** |
|  | Female | WT | 7 | 81.4 ± 12 | 393 ± 50 | 277 ± 38 | 179 ± 18 | 143 ± 12 |
|  | Female | *Mest*^pKO^ | 6 | 80.2 ± 9.3 | 370 ± 89 | 211 ± 32 | 148 ± 26 | 112 ± 17 |
| **Pvalue=** |  |  |  | 0.84 | 0.56 | **0.0058** | **0.028** | **0.0026** |

Data in **Panel A** shows the amount of variation (%) contributed by time and genotype (wildtype, WT; Mest**^pko^**, pko) for longitudinal measurements of phenotypic changes in BW, fat mass and lean mass in mice fed either control diet (CD) or Western diet (WD). Data in Panel A were analyzed using 2W-ANOVA and p-values are indicated in the table. **Panel B** shows values of blood glucose at time points from 0 to 120 min after glucose injection. Statistical analyses was performed via simple t-test between blood glucose values for each genotype at each time point. A p-value of ≤ 0.05 is considered significant.
