# Supplementary material for: Increased spontaneous physical activity in female MEST-deficient mice protects against diet-induced obesity
Source: Front Endocrinol (Lausanne). 2025 Oct 29;16:1680158. doi: 10.3389/fendo.2025.1680158 (PMC12609188; doi:10.3389/fendo.2025.1680158)
Supplement: Supplementary file 9 [file Table2.docx]

**Table S2A**

**Statistical analysis of indirect calorimetry data (144 hr; D5 to D10) for 18 wk old WT and *Mest*^pko^ female mice fed control diet for 10 wk**

| **Generalized Linear Models (*p* values); CD** | | | | | | | | | |
| --- | --- | --- | --- | --- | --- | --- | --- | --- | --- |
|  | **24-hour** | | | **Light** | | | **Dark** | | |
| **Effect** | **BW** | **GT** | **BW x GT** | **BW** | **GT** | **BW x GT** | **BW** | **GT** | **BW x GT** |
| *Energy Expenditure (kcal/period)* | **0.040** | **0.084** | >0.10 | >0.10 | >0.10 | >0.10 | **0.0021** | **0.011** | >0.10 |
| *Oxygen Consumption (ml/hr)* | **0.034** | **0.079** | >0.10 | >0.10 | >0.10 | >0.10 | **0.0016** | **0.011** | >0.10 |
| *Carbon Dioxide Production (ml/hr)* | **0.085** | >0.10 | >0.10 | >0.10 | >0.10 | >0.10 | **0.0074** | **0.013** | >0.10 |
|  |  |  |  |  |  |  |  |  |  |
|  | **LM** | **GT** | **LM x GT** | **LM** | **GT** | **LM x GT** | **LM** | **GT** | **LM x GT** |
| *Energy Expenditure (kcal/period)* | >0.10 | >0.10 | >0.10 | >0.10 | >0.10 | >0.10 | **0.018** | >0.10 | >0.10 |
| *Oxygen Consumption (ml/hr)* | >0.10 | >0.10 | >0.10 | >0.10 | >0.10 | >0.10 | **0.017** | >0.10 | >0.10 |
| *Carbon Dioxide Production (ml/hr)* | >0.10 | >0.10 | >0.10 | >0.10 | >0.10 | >0.10 | **0.026** | **0.066** | >0.10 |
|  |  |  |  |  |  |  |  |  |  |
|  | **FM** | **GT** | **FM x GT** | **FM** | **GT** | **FM x GT** | **FM** | **GT** | **FM x GT** |
| *Energy Expenditure (kcal/period)* | >0.10 | >0.10 | >0.10 | >0.10 | >0.10 | >0.10 | >0.10 | >0.10 | >0.10 |
| *Oxygen Consumption (ml/hr)* | >0.10 | >0.10 | >0.10 | >0.10 | >0.10 | >0.10 | >0.10 | >0.10 | >0.10 |
| *Carbon Dioxide Production (ml/hr)* | >0.10 | >0.10 | >0.10 | >0.10 | >0.10 | >0.10 | >0.10 | >0.10 | >0.10 |

| **ANOVA (*p* values for genotype effect)** | | | |
| --- | --- | --- | --- |
|  | **24-hour** | **Light** | **Dark** |
| *Respiratory Exchange Ratio* | >0.10 | >0.10 | **0.016** |
| *Pedestrian Locomotion (m)* | **0.032** | **0.031** | **0.034** |
| *Total Distance in Cage (m)* | **0.023** | **0.020** | **0.029** |
| *Locomotor Activity (beam breaks)* | >0.10 | >0.10 | >0.10 |

P-values obtained using generalized linear modeling with BW, lean mass (LM) and fat mass (FM) as covariates. One-way ANOVA p-values obtained for mass-independent variables. Data analyzed using CalR. Data were collected for 144 h (D5 to D10). P-values <0.05 are highlighted in bold. GT, genotype.

**Table S2B**

**Statistical analysis of indirect calorimetry data (72 hr, D2 to D5) for 19 wk old WT and *Mest*^pko^ female mice fed Western diet for 11 wk**

| **Generalized Linear Models (*p* values); CD** | | | | | | | | | |
| --- | --- | --- | --- | --- | --- | --- | --- | --- | --- |
|  | **24-hour** | | | **Light** | | | **Dark** | | |
| **Effect** | **BW** | **GT** | **BW x GT** | **BW** | **GT** | **BW x GT** | **BW** | **GT** | **BW x GT** |
| *Energy Expenditure (kcal/period)* | >0.10 | >0.10 | >0.10 | **0.099** | >0.10 | >0.10 | >0.10 | >0.10 | >0.10 |
| *Oxygen Consumption (ml/hr)* | >0.10 | >0.10 | >0.10 | >0.10 | >0.10 | >0.10 | >0.10 | >0.10 | >0.10 |
| *Carbon Dioxide Production (ml/hr)* | >0.10 | >0.10 | >0.10 | **0.071** | >0.10 | >0.10 | >0.10 | >0.10 | >0.10 |
|  |  |  |  |  |  |  |  |  |  |
|  | **LM** | **GT** | **LM x GT** | **LM** | **GT** | **LM x GT** | **LM** | **GT** | **LM x GT** |
| *Energy Expenditure (kcal/period)* | **0.044** | >0.10 | >0.10 | **0.023** | >0.10 | >0.10 | >0.10 | >0.10 | >0.10 |
| *Oxygen Consumption (ml/hr)* | **0.047** | >0.10 | >0.10 | **0.025** | >0.10 | >0.10 | >0.10 | >0.10 | >0.10 |
| *Carbon Dioxide Production (ml/hr)* | **0.035** | >0.10 | >0.10 | **0.016** | >0.10 | >0.10 | >0.10 | >0.10 | >0.10 |
|  |  |  |  |  |  |  |  |  |  |
|  | **FM** | **GT** | **FM x GT** | **FM** | **GT** | **FM x GT** | **FM** | **GT** | **FM x GT** |
| *Energy Expenditure (kcal/period)* | >0.10 | >0.10 | >0.10 | >0.10 | >0.10 | >0.10 | >0.10 | >0.10 | >0.10 |
| *Oxygen Consumption (ml/hr)* | >0.10 | >0.10 | >0.10 | >0.10 | >0.10 | >0.10 | >0.10 | >0.10 | >0.10 |
| *Carbon Dioxide Production (ml/hr)* | >0.10 | >0.10 | >0.10 | >0.10 | >0.10 | >0.10 | >0.10 | >0.10 | >0.10 |

| **ANOVA (*p* values for genotype effect)** | | | |
| --- | --- | --- | --- |
|  | **24-hour** | **Light** | **Dark** |
| *Respiratory Exchange Ratio* | >0.10 | >0.10 | >0.10 |
| *Pedestrian Locomotion (m)* | **<0.001** | **<0.001** | **<0.001** |
| *Total Distance in Cage (m)* | **<0.001** | **<0.001** | **<0.001** |
| *Locomotor Activity (beam breaks)* | **0.0090** | **0.0081** | **0.016** |

P-values obtained using generalized linear modeling with BW, lean mass (LM) and fat mass (FM) as covariates. One-way ANOVA p-values obtained for mass-independent variables. Data analyzed using CalR. Data were collected for 72 h (D2 to D5). P-values <0.05 are highlighted in bold. GT, genotype.
