# Supplementary material for: Increased spontaneous physical activity in female MEST-deficient mice protects against diet-induced obesity
Source: Front Endocrinol (Lausanne). 2025 Oct 29;16:1680158. doi: 10.3389/fendo.2025.1680158 (PMC12609188; doi:10.3389/fendo.2025.1680158)
Supplement: Supplementary file 10 [file Table3.docx]

**Table S3A**

**Statistical analysis of indirect calorimetry data (168 hr; D2 to D9) for 12 wk old WT and *Mest*^pko^ male mice fed control diet for 4 wk**

| **Generalized Linear Models (*p* values); CD** | | | | | | | | | |
| --- | --- | --- | --- | --- | --- | --- | --- | --- | --- |
|  | **24-hour** | | | **Light** | | | **Dark** | | |
| **Effect** | **BW** | **GT** | **BW x GT** | **BW** | **GT** | **BW x GT** | **BW** | **GT** | **BW x GT** |
| *Energy Expenditure (kcal/period)* | >0.10 | >0.10 | >0.10 | >0.10 | >0.10 | >0.10 | >0.10 | >0.10 | >0.10 |
| *Oxygen Consumption (ml/hr)* | >0.10 | >0.10 | >0.10 | >0.10 | >0.10 | >0.10 | >0.10 | >0.10 | >0.10 |
| *Carbon Dioxide Production (ml/hr)* | >0.10 | >0.10 | >0.10 | >0.10 | >0.10 | >0.10 | >0.10 | >0.10 | >0.10 |
|  |  |  |  |  |  |  |  |  |  |
|  | **LM** | **GT** | **LM x GT** | **LM** | **GT** | **LM x GT** | **LM** | **GT** | **LM x GT** |
| *Energy Expenditure (kcal/period)* | >0.10 | >0.10 | >0.10 | >0.10 | >0.10 | >0.10 | >0.10 | >0.10 | >0.10 |
| *Oxygen Consumption (ml/hr)* | >0.10 | >0.10 | >0.10 | >0.10 | >0.10 | >0.10 | >0.10 | >0.10 | >0.10 |
| *Carbon Dioxide Production (ml/hr)* | >0.10 | >0.10 | >0.10 | >0.10 | >0.10 | >0.10 | >0.10 | >0.10 | >0.10 |
|  |  |  |  |  |  |  |  |  |  |
|  | **FM** | **GT** | **FM x GT** | **FM** | **GT** | **FM x GT** | **FM** | **GT** | **FM x GT** |
| *Energy Expenditure (kcal/period)* | 0.061 | >0.10 | >0.10 | >0.10 | >0.10 | >0.10 | **0.014** | >0.10 | >0.10 |
| *Oxygen Consumption (ml/hr)* | 0.064 | >0.10 | >0.10 | >0.10 | >0.10 | >0.10 | **0.017** | >0.10 | >0.10 |
| *Carbon Dioxide Production (ml/hr)* | 0.053 | >0.10 | >0.10 | >0.10 | >0.10 | >0.10 | **0.009** | >0.10 | >0.10 |

| **ANOVA (*p* values for genotype effect)** | | | |
| --- | --- | --- | --- |
|  | **24-hour** | **Light** | **Dark** |
| *Respiratory Exchange Ratio* | >0.10 | >0.10 | >0.10 |
| *Pedestrian Locomotion (m)* | >0.10 | >0.10 | >0.10 |
| *Total Distance in Cage (m)* | >0.10 | >0.10 | >0.10 |
| *Locomotor Activity (beam breaks)* | >0.10 | >0.10 | >0.10 |

P-values obtained using generalized linear modeling with BW, lean mass (LM) and fat mass (FM) as covariates. One-way ANOVA p-values obtained for mass-independent variables. Data analyzed using CalR. Data were collected for 168 h (D2 to D9). P-values <0.05 are highlighted in bold. GT, genotype.

**Table S3B**

**Statistical analysis of indirect calorimetry data (168 hr, D2 to D9) for 12 wk old WT and *Mest*^pko^ male mice fed Western diet for 4 wk**

| **Generalized Linear Models (*p* values); WD** | | | | | | | | | |
| --- | --- | --- | --- | --- | --- | --- | --- | --- | --- |
|  | **24-hour** | | | **Light** | | | **Dark** | | |
| **Effect** | **BW** | **GT** | **BW x GT** | **BW** | **GT** | **BW x GT** | **BW** | **GT** | **BW x GT** |
| *Energy Expenditure (kcal/period)* | >0.10 | >0.10 | >0.10 | **0.099** | >0.10 | >0.10 | >0.10 | >0.10 | >0.10 |
| *Oxygen Consumption (ml/hr)* | >0.10 | >0.10 | >0.10 | >0.10 | >0.10 | >0.10 | >0.10 | >0.10 | >0.10 |
| *Carbon Dioxide Production (ml/hr)* | >0.10 | >0.10 | >0.10 | **0.071** | >0.10 | >0.10 | >0.10 | >0.10 | >0.10 |
|  |  |  |  |  |  |  |  |  |  |
|  | **LM** | **GT** | **LM x GT** | **LM** | **GT** | **LM x GT** | **LM** | **GT** | **LM x GT** |
| *Energy Expenditure (kcal/period)* | 0.095 | 0.060 | >0.10 | >0.10 | 0.070 | >0.10 | 0.093 | 0.064 | >0.10 |
| *Oxygen Consumption (ml/hr)* | >0.10 | 0.063 | >0.10 | 0.081 | 0.081 | >0.10 | 0.094 | 0.060 | >0.10 |
| *Carbon Dioxide Production (ml/hr)* | 0.067 | **0.050** | >0.10 | 0.061 | **0.038** | >0.10 | >0.10 | 0.092 | >0.10 |
|  |  |  |  |  |  |  |  |  |  |
|  | **FM** | **GT** | **FM x GT** | **FM** | **GT** | **FM x GT** | **FM** | **GT** | **FM x GT** |
| *Energy Expenditure (kcal/period)* | >0.10 | >0.10 | >0.10 | >0.10 | >0.10 | >0.10 | >0.10 | >0.10 | >0.10 |
| *Oxygen Consumption (ml/hr)* | >0.10 | >0.10 | >0.10 | >0.10 | >0.10 | >0.10 | >0.10 | >0.10 | >0.10 |
| *Carbon Dioxide Production (ml/hr)* | >0.10 | >0.10 | >0.10 | >0.10 | >0.10 | >0.10 | >0.10 | >0.10 | >0.10 |

| **ANOVA (*p* values for genotype effect)** | | | |
| --- | --- | --- | --- |
|  | **24-hour** | **Light** | **Dark** |
| *Respiratory Exchange Ratio* | >0.10 | >0.10 | >0.10 |
| *Pedestrian Locomotion (m)* | >0.10 | >0.10 | >0.10 |
| *Total Distance in Cage (m)* | >0.10 | >0.10 | >0.10 |
| *Locomotor Activity (beam breaks)* | >0.10 | >0.10 | >0.10 |

P-values obtained using generalized linear modeling with BW, lean mass (LM) and fat mass (FM) as covariates. One-way ANOVA p-values obtained for mass-independent variables. Data analyzed using CalR. Data were collected for 168 h (D2 to D9). P-values <0.05 are highlighted in bold. GT, genotype.
