# Supplementary material for: Increased spontaneous physical activity in female MEST-deficient mice protects against diet-induced obesity
Source: Front Endocrinol (Lausanne). 2025 Oct 29;16:1680158. doi: 10.3389/fendo.2025.1680158 (PMC12609188; doi:10.3389/fendo.2025.1680158)
Supplement: Supplementary file 12 [file Table5.docx]

**Table S4**

**WAT gene expression: Analyses of female mice fed CD or WD using two-way ANOVA**

**Panel A**

|  |  |  | **Control Diet (CD)** | | **Western Diet (WD)** | | **2W-ANOVA (% of total variation)** | | |
| --- | --- | --- | --- | --- | --- | --- | --- | --- | --- |
| **Gene** | **Tissue** | **Sex** | **WT (n)** | **pKO (n)** | **WT (n)** | **pKO (n)** | **Diet** | **Genotype** | **Interaction** |
| Mest | iWAT | F | 8 | 7 | 6 | 7 | 27.3**^d^** | 33.8**^d^** | 27.3**^d^** |
| Mest | gWAT | F | 8 | 8 | 6 | 7 | 29.3**^d^** | 40.7**^d^** | 29.3**^d^** |
| Klf14 | iWAT | F | 8 | 8 | 6 | 7 | 45.2**^d^** | 18.2**^c^** | 10.0**^a^** |
| Klf14 | gWAT | F | 8 | 8 | 6 | 7 | 36.5**^d^** | 24.9**^c^** | 11.6**^c^** |
| Lep | iWAT | F | 8 | 8 | 6 | 7 | 40.5**^d^** | 20.8**^c^** | 17.9**^c^** |
| Lep | gWAT | F | 8 | 8 | 6 | 7 | 48.2**^d^** | 22.28**^d^** | 21.2**^d^** |
| Ucp1 | iWAT | F | 8 | 8 | 6 | 7 | 18.6**^b^** | 26.4**^c^** | 17.8**^b^** |
| Ucp1 | gWAT | F | 8 | 8 | 6 | 7 | 16.4**^b^** | 23.7**^b^** | 13.2**^a^** |
| Tfam | iWAT | F | 8 | 8 | 6 | 7 | 96.4**^d^** | 0.075 | 0.038 |
| Tfam | gWAT | F | 8 | 8 | 6 | 7 | 95.1**^d^** | 0.010 | 0.032 |
| Cpt1b | iWAT | F | 8 | 8 | 6 | 7 | 74.8**^d^** | 0.72 | 0.064 |
| Cpt1b | gWAT | F | 8 | 8 | 6 | 7 | 63.3**^d^** | 1.82 | 0.047 |
| Dio2 | iWAT | F | 8 | 8 | 6 | 7 | 3.22 | 33.4**^b^** | 3.19 |
| Dio2 | gWAT | F | 8 | 8 | 6 | 7 | 0.52 | 3.00 | 13.4 |
| DIO2 Act | iWAT | F | 8 | 8 | 6 | 7 | 52.8**^d^** | 4.89 | 0.55 |
| DIO2 Act | gWAT | F | 8 | 8 | 6 | 7 | 67.5**^d^** | 4.62**^a^** | 1.83 |

**Panel B**

|  |  |  | **iWAT** | | **gWAT** | | **2W-ANOVA (% of total variation)** | | |
| --- | --- | --- | --- | --- | --- | --- | --- | --- | --- |
| **Gene** | **Diet** | **Sex** | **WT (n)** | **pKO (n)** | **WT (n)** | **pKO (n)** | **WAT depot** | **Genotype** | **Interaction** |
| Ppargc1a | WD | F | 6 | 7 | 6 | 7 | 0.038 | 53.3**^d^** | 2.47 |
| Cidea | WD | F | 6 | 7 | 6 | 7 | 8.32 | 35.5**^d^** | 2.90 |
| Ppara | WD | F | 6 | 7 | 6 | 7 | 20.7**^b^** | 27.2**^b^** | 0.55 |
| Gpd2 | WD | F | 6 | 7 | 6 | 7 | 11.7**^a^** | 36.2**^c^** | 2.53 |

**Panel A** shows the variation (%) in the expression of genes in inguinal and gonadal white adipose tissue (iWAT and gWAT) that is contributed by diet (control diet, CD; Western diet, WD) or genotype (wildtype, WT; *Mest***^pko^**, pko). **Panel B** shows % variation of gene expression explained by WAT depot and genotype. Analyses was performed using 2W-ANOVA. Data annotated with the superscripts a, b, c and d indicate p-values of <0.05. <0.01, <0.001 and 0.0001 respectively.
